# Graphene nanoribbon-based supramolecular ensembles with dual-receptor targeting function for targeted photothermal tumor therapy†

**DOI:** 10.1039/d1sc02154k

**Published:** 2021-07-15

**Authors:** Wei-Tao Dou, Fugui Xu, Chen-Xi Xu, Jie Gao, Hong-Bo Ru, Xiangfeng Luan, Jiacheng Zhang, Ling Zhu, Adam C. Sedgwick, Guo-Rong Chen, Yi Zang, Tony D. James, He Tian, Jia Li, Yiyong Mai, Xiao-Peng He

**Affiliations:** Key Laboratory for Advanced Materials and Joint International Research Laboratory of Precision Chemistry and Molecular Engineering, Feringa Nobel Prize Scientist Joint Research Center, School of Chemistry and Molecular Engineering, Frontiers Center for Materiobiology and Dynamic Chemistry, East China University of Science and Technology 130 Meilong RD Shanghai 200237 P. R. China xphe@ecust.edu.cn; School of Chemistry and Chemical Engineering, Frontiers Science Center for Transformative Molecules, Shanghai Key Laboratory of Electrical Insulation and Thermal Ageing, Shanghai Jiao Tong University 800 Dongchuan RD Shanghai 200240 P. R. China mai@sjtu.edu.cn; National Center for Drug Screening, State Key Laboratory of Drug Research, Shanghai Institute of Materia Medica, Chinese Academy of Sciences 189 Guo Shoujing Rd. Shanghai 201203 P. R. China jli@simm.ac.cn; Department of Chemistry, The University of Texas at Austin Austin Texas 78712-1224 USA; Department of Chemistry, University of Bath Bath BA2 7AY UK; School of Chemistry and Chemical Engineering, Henan Normal University Xinxiang 453007 China

## Abstract

Triple negative breast cancer (TNBC) is one of the most malignant subtypes of breast cancer. Here, we report the construction of graphene nanoribbon (GNR)-based supramolecular ensembles with dual-receptor (mannose and α_v_β_3_ integrin receptors) targeting function, denoted as **GNR-Man/PRGD**, for targeted photothermal treatment (PTT) of TNBC. The **GNR-Man/PRGD** ensembles were constructed through the solution-based self-assembly of mannose-grafted GNRs (**GNR-Man**) with a pyrene-tagged α_v_β_3_ integrin ligand (**PRGD**). Enhanced PTT efficacies were achieved both *in vitro* and *in vivo* compared to that of the non-targeting equivalents. Tumor-bearing live mice were administered (tail vein) with **GNR-Man/PRGD** and then each mice group was subjected to PTT. Remarkably, **GNR-Man/PRGD** induced complete ablation of the solid tumors, and no tumor regrowth was observed over a period of 15 days. This study demonstrates a new and promising platform for the development of photothermal nanomaterials for targeted tumor therapy.

## Abstract 2

Dual receptor-targeting supramolecular glycomaterials are constructed based on graphene nanoribbons for the targeted photothermal therapy of triple-negative breast cancer *in vivo*.

## Graphical abstract

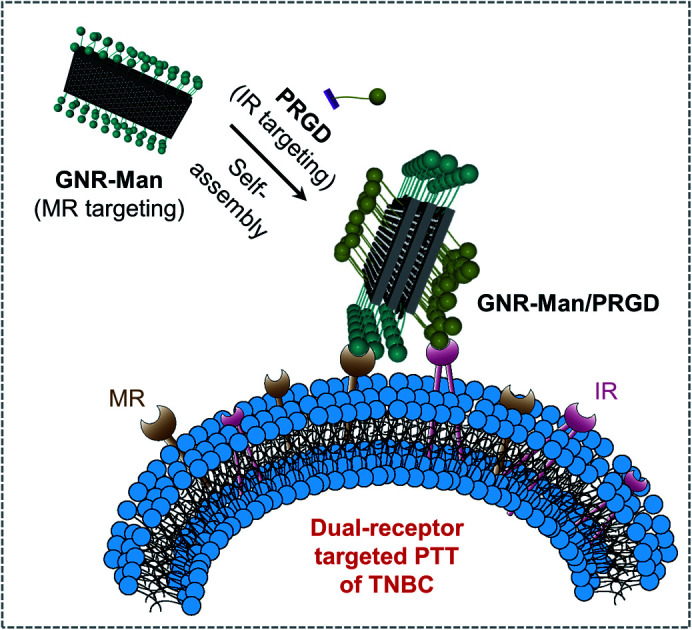

## Introduction

Triple-negative breast cancer (TNBC) is an aggressive subtype of breast cancer associated with poor patient prognosis. This is due to its high tumor heterogeneity, rapid proliferation and early metastasis.^1,2^ TNBC lacks the expression of estrogen receptor (ER), progesterone receptor (PR) and human epidermal growth factor receptor 2 (HER2),^3,4^ which eliminates the possibility of using targeted therapies. This necessitates the use of traditional chemotherapy, radiotherapy, and surgical intervention.^5–8^ Therefore, new and effective therapeutic strategies are needed to overcome the limitations/challenges (non-invasive and non-toxic) for current treatments of TNBC.^9,10^

In recent years, photothermal therapy (PTT) has emerged as an attractive light-based therapeutic approach for the treatment of diseases.^11–15^ In brief, PTT involves the light irradiation of an optical absorber (PTT agent) that is localised at the diseased location. This results in the conversion of light energy to heat, which then induces cell death.^16–18^ In comparison to traditional therapeutics, the use of light affords a non-invasive strategy with high specificity.^19–23^ To date, several platforms have been developed for PTT and were demonstrated in a range of disease models.^24–28^ Graphene nanoribbons (GNRs) have displayed significant photothermal conversion efficiencies (PCE) for potential use as PTT.^29–36^ However, targeted biocompatible platforms are needed for *in vivo* applications to limit off-target toxicities and to improve therapeutic outcomes.

Throughout nature, glycoconjugates are known to selectively bind to carbohydrate receptors and modulate a variety of physiological and pathological processes. Such examples include cell–cell recognition,^37^ signal transmission,^38^ virus invasion^39^ and bacterial infection.^40^ Previous studies have reported the overexpression of mannose receptors (MRs) on the cell surface of tumor-associated macrophages (TAMs). TAMs have been associated with the regulation of immunity, cancer metastasis and progression of ovarian and breast cancer.^41^ In recent years, researchers have exploited the high affinity of mannose for MRs to develop targeted imaging and therapeutic agents.^42,43^ These efforts inspired our group to develop glycoconjugate-based materials (abbreviated as glycomaterials) for targeted PTT of TNBC both *in vitro* and *in vivo*.

## Results and discussion

**Scheme 1:**
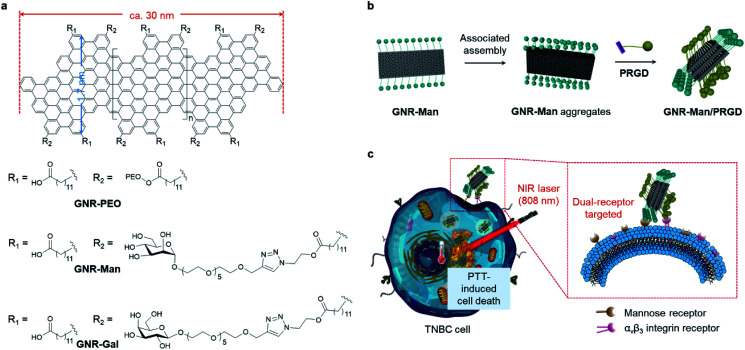
(a) Structures of **GNR-PEO**, **GNR-Gal** and **GNR-Man**. The GNR backbone possesses a well-defined structure with an arm-chair edge architecture, a uniform width of 1.7 nm and an average length of 30 nm. (b) Schematic illustration of the supramolecular self-assembly of **GNR-Man** with a pyrenyl peptide ligand (**PRGD**) in aqueous solution to produce **GNR-Man/PRGD** ensembles. (c) Targeted photothermal treatment of TNBC cells through dual-interaction of **GNR-Man/PRGD** with both MR and α_v_β_3_ integrin receptors under NIR light irradiation.

Here, we report the construction of two glycomaterials (**GNR-Man** and **GNR-Gal**), utilizing our previously reported carboxyl-functionalized GNR (**GNR-COOH**) precursor (Scheme 1a and S2†).^44,45^**GNR-Man** and **GNR-Gal** were synthesized by grafting mannose (grafting percentage 72%) and galactose (grafting percentage 59%) at the edge of GNRs for use as receptor-targeting agents, respectively. Both **GNR-Man** and **GNR-Gal** glycomaterials achieved targeted PTT of TNBC (MDA-MB-231) and hepatoma (Hep-G2) cell lines, which overexpress MRs and asialoglycoprotein receptors (ASGPr – galactose selective), respectively (Scheme 1c). We then explored, the supramolecular self-assembly between **GNR-Man** and a pyrene-tagged peptide ligand, **PRGD** (pyrene-KKKRGD, a well-known α_v_β_3_ integrin-targeting ligand),^46^ to generate GNR-based supramolecular ensembles with dual-receptor targeting function (denoted as **GNR-Man/PRGD**) (Scheme 1b), which were employed for the precision-enhanced PTT of MDA-MB-231 (a TNBC cell line)-xenograft mice (tail-vein injection).

To the best of our knowledge, this study represents the first example of structurally defined GNRs for targeted tumor therapy. Previously, GNRs have been shown to have a superior photothermal-conversion efficiency exceeding those of many photothermal materials commonly used for PTT.^29,30^ However, these systems were not designed for biological application. In this work, to avoid off-target toxicity and realize tumor specificity, we have aimed to modify structurally defined GNRs with both carbohydrate and peptide ligands to produce dual-targeting **GNR-Man/PRGD** supramolecular ensembles for the PTT treatment of tumors. Using **GNR-Man/PRGD** as an *in vivo* PTT agent, complete ablation of TNBC-xenograft solid tumors was achieved, with no tumor regrowth over a period of 15 days. In addition, immunohistochemical assays suggested good biocompatibility of the **GNR-Man/PRGD** ensembles.

Carboxyl-functionalized GNRs (**GNR-COOH**) and poly(ethylene oxide) (PEO)-functionalized (molecular weight = 2000 g mol^−1^) GNR (**GNR-PEO**, grafting percentage 42%) were synthesized according to previously reported protocols;^44,45^**GNR-PEO** was used as a control sample. GNRs functionalized with azido units (**GNR-N3**) were synthesized through the esterification of **GNR-COOH** with 2-azidoethanol. Subsequently, the Cu(i)-catalyzed azide–alkyne “click reaction” between **GNR-N3** and *O*-alkynyl-galactoside/*O*-alkynyl-mannoside afforded **GNR-Gal** and **GNR-Man**, respectively (Schemes S1 and S2†). All functionalized GNRs were characterized by Fourier transform infrared (FTIR) spectroscopy (Fig. S2†), and the grafting degrees of **GNR-Gal** and **GNR-Man** were determined using thermal gravimetric analysis (TGA) (Fig. S1†). Based on the weight loss at 600 °C in the TGA curves, the grafting percentages of **GNR-Gal** and **GNR-Man** were estimated to be 59% and 72%, respectively.

**Fig. 1:**
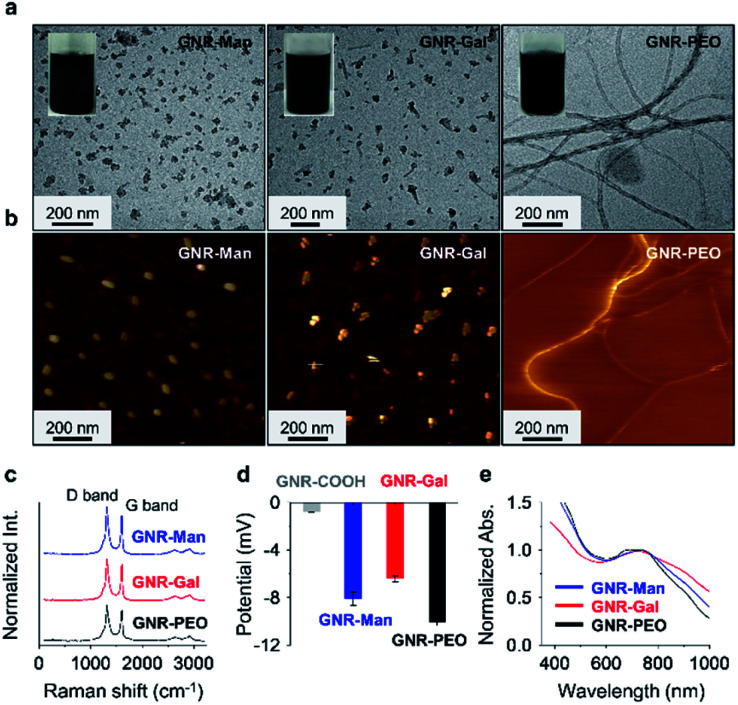
(a) Transmission electron microscopy (TEM) and (b) atomic force microscopy (AFM) images of the supramolecular assemblies formed by **GNR-Man**, **GNR-Gal** and **GNR-PEO** in aqueous phase. (c) Raman spectra, (d) zeta potential and (e) UV-vis-NIR absorption spectra of **GNR-Man**, **GNR-Gal** and **GNR-PEO**. The concentration of the GNRs used for the TEM, Raman, zeta potential and UV-vis-NIR absorption measurements was 50 μg mL^−1^, and that for the AFM measurement was 2.5 μg mL^−1^.

The sonication of **GNR-Gal**, **GNR-Man** or **GNR-PEO** in a phosphate buffer saline (PBS) solution (0.01 M, pH 7.40, 0.5% Triton X-100 (TX-100), v/v) afforded their stable homogeneous dispersions without visible precipitation (Fig. 1a). Transmission electron microscopy (TEM) and atomic force microscopic (AFM) images indicated that **GNR-Man** and **GNR-Gal** formed ensembles with an average diameter of *ca.* 35 nm and 40 nm, respectively (Fig. 1a and b). In contrast, **GNR-PEO** formed spring-like nanowires with an average diameter of 20 nm and a length up to micrometer scale (Fig. 1a and b).^29^ The hydrodynamic diameters of **GNR-Man** and **GNR-Gal** were determined to be 61 nm and 86 nm by dynamic light scattering (DLS), respectively (Fig. S3†). The larger particle diameters determined by DLS than those by TEM are attributed to stretching of the alkyl and oligo(ethylene oxide) chains in liquid phase. Typical Raman signals corresponding to the D band (disordered band) at 1325 cm^−1^ and the G band (graphite band) at 1603 cm^−1^ were consistently observed for **GNR-PEO**, **GNR-Gal** and **GNR-Man**.^47^ This suggests that the introduction of the glycoligands did not affect the conjugated GNR backbone (Fig. 1c).^30^ In addition, the modification with hydrophilic glycosyl and PEO groups significantly improved water solubility of the GNRs with respect to **GNR-COOH**, which is supported by zeta potential analysis (Fig. 1d). The good aqueous dispersibility of the GNRs is reflected in a broad-band UV-vis-NIR adsorption ranging from 400 to 1000 nm (Fig. 1e).

**Fig. 2:**
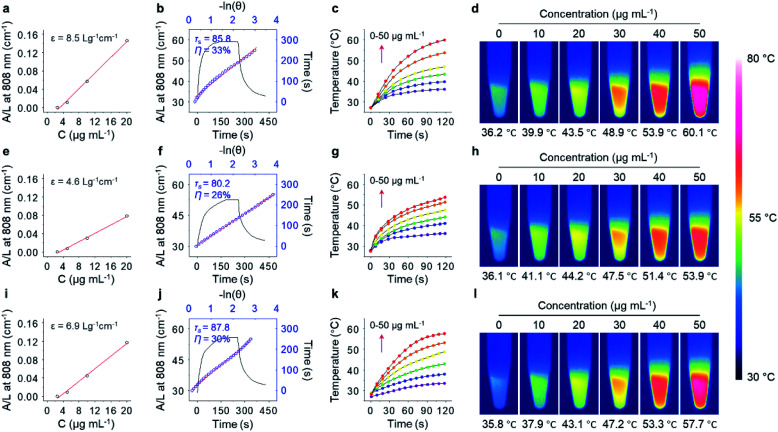
Mass extinction coefficient of (a) **GNR-Man**, (e) **GNR-Gal** and (i) **GNR-PEO** at 808 nm. Photothermal-conversion efficiency (PCE) of (b) **GNR-Man** (50 μg mL^−1^), (f) **GNR-Gal** (50 μg mL^−1^) and (j) **GNR-PEO** (50 μg mL^−1^); the blank curve is the heating and cooling process of the materials within 450 s under NIR light irradiation (808 nm, 1.0 W cm^−2^, 2 min), and the blue dots were fitted to calculate the time constant (*τ*_s_) and PCE (*η*). Time-dependent temperature changes (0, 10, 20, 30, 40 and 50 μg mL^−1^) and thermal images of aqueous dispersions of (c, d) **GNR-Man**, (g, h) **GNR-Gal** and (k, l) **GNR-PEO** under NIR light irradiation (808 nm, 1.0 W cm^−2^) for 2 min in PBS (0.01 M, pH 7.40, 0.5% TX-100, v/v).

With the excellent water dispersibility and inherent near-infrared (NIR) absorption characteristics (Fig. S4†), the mass extinction coefficient and PCE of the three GNRs under NIR light irradiation (808 nm, 1.0 W cm^−2^) was determined. The extinction coefficient of **GNR-Man** (Fig. 2a), **GNR-Gal** (Fig. 2e) and **GNR-PEO** (Fig. 2i) at 808 nm was measured to be 8.5 L g^−1^ cm^−1^, 4.6 L g^−1^ cm^−1^ and 6.9 L g^−1^ cm^−1^, respectively. The PCE of **GNR-Man** (Fig. 2b), **GNR-Gal** (Fig. 2f) and **GNR-PEO** (Fig. 2j) at 808 nm was determined to be 33%, 26% and 30%, respectively.^48^ The photothermal performance of the GNRs in PBS at increasing concentrations (0, 10, 20, 30, 40 and 50 μg mL^−1^) were evaluated (808 nm, 1.0 W cm^−2^, 2 min), which demonstrated a concentration-dependent photothermal performance (Fig. 2c, g and k for **GNR-Man**, **GNR-Gal** and **GNR-PEO**, respectively). Photothermal imaging indicated that the highest temperature reached was 60.1 °C for a **GNR-Man** containing solution (50 μg mL^−1^) (Fig. 2d). While, the highest temperature reached was 53.9 °C and 57.7 °C for **GNR-Gal** (Fig. 2h) and **GNR-PEO** (Fig. 2i), respectively. No obvious change in the photothermal performance was observed for **GNR-Man** after ten cycles (Fig. S5†). The above results confirmed the potential of the GNRs as PTT agents.

**Fig. 3:**
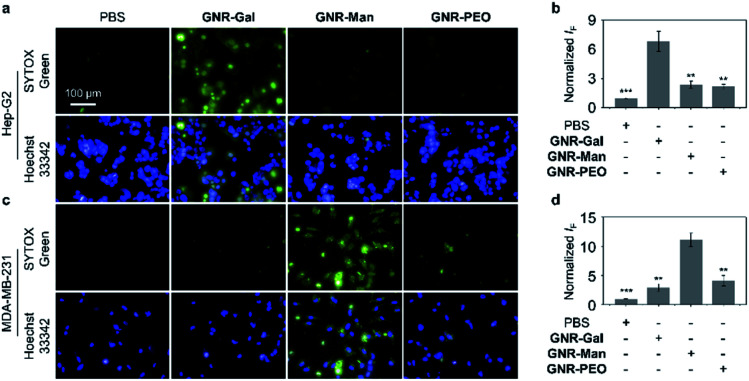
Fluorescence imaging and quantification of dead cells of (a, b) Hep-G2 (**P < 0.01 and ***P < 0.001 with respect to the **GNR-Gal** group) and (c, d) MDA-MB-231 (**P < 0.01 and ***P < 0.001 with respect to the **GNR-Man** group) incubated with **GNR-Gal**, **GNR-Man** and **GNR-PEO** (concentration: 40 μg mL^−1^) under NIR light irradiation (808 nm, 1.0 W cm^−2^) for 3 min. The scale bar is applicable to all the images. Dead and total cells were stained with SYTOX Green (*λ*_ex_ = 490–510 nm, *λ*_em_ = 530–590 nm) and Hoechst 33342 (*λ*_ex_ = 360–400 nm, *λ*_em_ = 410–480 nm), respectively. S. D. means standard deviation (*n* = 3).

The targeting ability of the glycosylated GNRs were subsequently evaluated using a cellular assay. Hep-G2 (human hepatoma) and MDA-MB-231 (TNBC) cell lines were used as they overexpress ASGPrs (galactose-selective receptor) and MRs (mannose-selective receptor), respectively. The corresponding receptor expression was confirmed by real-time quantitative polymerase chain reaction (Fig. S6†). Cell viability study without light irradiation was performed for each GNR sample, in which minimal cytotoxicity for materials ranging from 0 to 40 μg mL^−1^ was observed. (Fig. S7†). To illustrate the PTT efficacy, a concentration of 40 μg mL^−1^ was used for cellular experiments. The targeting ability of each GNR after light irradiation (808 nm, 1.0 W cm^−2^, 3 min) was evaluated by quantifying the number of dead cells using fluorescence imaging with SYTOX Green, as shown in Fig. 3, **GNR-PEO** displayed a minimal phototoxicity for each cell line. In contrast, treatment of the appropriate cell lines with **GNR-Gal** and **GNR-Man** (**GNR-Gal** – Hep-G2; **GNR-Man** – MDA-MB-231) followed by light irradiation led to a 3.1-fold and 2.7-fold increase in dead cells, respectively, in comparison to that of **GNR-PEO** (Fig. 3a–d). Minimal dead cells were observed for **GNR-Gal** treated MDA-MB-231 cells and **GNR-Man** treated Hep-G2 cells after light irradiation. These results illustrate the potential cellular targeting ability of the glycosylated GNRs.

The photothermal effect for both **GNR-Gal** and **GNR-Man** was dependent on the GNR concentration (Fig. S8†) and the laser power (Fig. S9†). The receptor-dependent targeting for the GNR-based glycomaterials was demonstrated *via* a competition assay. The pre-incubation of free galactose with Hep-G2 and the pre-incubation of free mannose with MDA-MB-231 reduced the photothermal therapeutic effect (decreased quantity of dead cells) of **GNR-Gal** and **GNR-Man** in a concentration-dependent manner, respectively (Fig. S10†). This further suggests that the targeted PTT effect observed is dependent on the glycoligand-receptor recognition. These preliminary results illustrate the success of our glycosylation strategy for enhancing the selective internalization of GNRs in a target cancer cell line. Given our focus was on developing a therapeutic platform for TNBC, **GNR-Man** was the main focus for the remainder of this study.

**Fig. 4:**
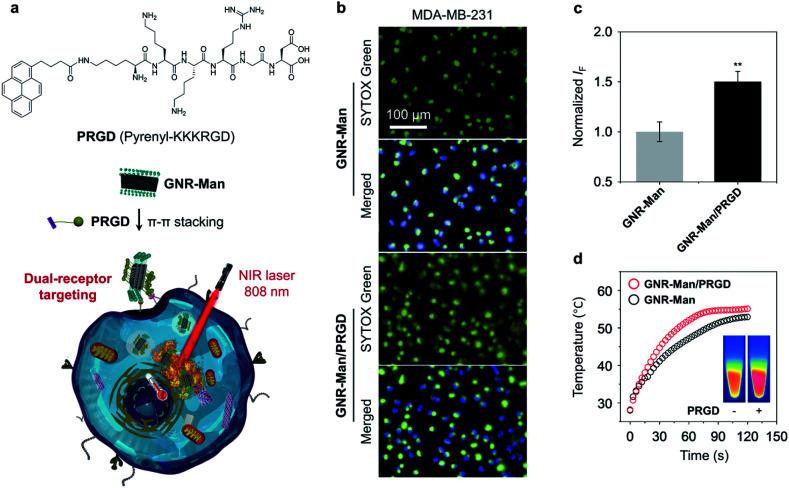
(a) Chemical structure of pyrene-functionalized RGD peptide (**PRGD**) and the basic schematic for the formation of **GNR-Man/PRGD**. (b) Fluorescence imaging and (c) quantification of dead cells of MDA-MB-231 that were incubated with either **GNR-Man** (40 μg mL^−1^) or **GNR-Man/PRGD** (40 μg mL^−1^/20 μM) followed by NIR light irradiation (808 nm, 1.0 W cm^−2^) for 3 min. (d) Time-dependent temperature increase (inset: thermal images of **GNR-Man** and **GNR-Man/PRGD**) of **GNR-Man** (40 μg mL^−1^) with and without **PRGD** (20 μM) under NIR light irradiation (808 nm, 1.0 W cm^−2^) for 3 min. Dead and total cells were stained using SYTOX Green (*λ*_ex_ = 490–510 nm, *λ*_em_ = 530–590 nm) and Hoechst 33342 (*λ*_ex_ = 360–400 nm, *λ*_em_ = 410–480 nm), respectively. **P < 0.01. S. D. means standard deviation (*n* = 3).

To further enhance the targeting capability of the GNRs, we incorporated a peptide ligand onto **GNR-Man** (pyrenyl-KKKRGD, denoted as **PRGD**). This ligand is selective for integrin α_v_β_3_ receptors, which are expressed on the surface of a range of cancer cells including breast cancer.^49^ This chosen peptide ligand was modified with a pyrene moiety to enable its association with the GNRs through π–π stacking; the chemical structure of the pyrene-functionalized peptide **PRGD** is shown in Fig. 4a. The supramolecular assembly between **GNR-Man** and **PRGD** was achieved through their mixing in aqueous solution, followed by sonication for 15 min. The resultant mannose/**PRGD** functionalized supramolecular assemblies (**GNR-Man/PRGD**) were designed to target both MR and α_v_β_3_ receptors on the surface of TNBC cells (Fig. 4a).

The supramolecular ensemble **GNR-Man/PRGD** was characterised by TEM, DLS and Raman spectroscopy. Particle-like structures were observed for **GNR-Man/PRGD** (Fig. S11a†), which are morphologically similar to that of **GNR-Man**. However, an obvious increase in the sizes of the particles was observed in comparison to that of the **GNR-Man** counterpart (Fig. S11b†). In the Raman spectra, the *I*_D_/*I*_G_ ratio of **GNR-Man** (1.2) increased to 1.3 after self-assembly with **PRGD**, supporting the association of the GNR with the peptide ligand (Fig. S12†).^50^ The PCE of **GNR-Man/PRGD** did not change compared to that of **GNR-Man** only (Fig. 4d). DLS analysis revealed smaller PDI values for **GNR-Man/PRGD** and **GNR-Gal/PRGD** when compared to **GNR-Man** and **GNR-Gal** (Fig. S13a†), which is indicative of an enhanced aqueous dispersibility. The absolute zeta potential values for **GNR-Man/PRGD** and **GNR-Gal/PRGD** were larger than that of **GNR-Man** and **GNR-Gal** (Fig. S13b†), which indicates an improved aqueous stability. Additional DLS analysis showed no changes to the hydrodynamic diameter of **GNR-Man/PRGD** and **GNR-Gal/PRGD** over the course of 7 days (Figs. S13c and d†). In contrast, the hydrodynamic diameters of **GNR-Man** and **GNR-Gal** were shown to increase gradually over time, suggesting their slow aggregation. Overall, this data suggests an improved aqueous stability and solubility for GNRs/**PRGD** assemblies.

With these ideal solution-based properties observed for **GNR-Man/PRGD**, we subsequently evaluated its PTT performance against MDA-MB-231 cells. Indeed, more dead cells were observed (determined *via* SYTOX green staining) for **GNR-Man/PRGD** treatment when compared to cells treated with **GNR-Man** only (Fig. 4b and c). These selective properties of **GNR-Man/PRGD** for MDA-MB-321 cells was further confirmed through its evaluation with additional cancer cell lines. These include Hela cells (human cervical cell line),^51^ MCF-7 cells (human breast cancer cell line)^52^ and Hep-G2 cells (human hepatoma cell line),^53^ all of which are reported to have a lower expression of the integrin α_v_β_3_ receptor. Both **GNR-Man/PRGD** and **GNR-Man** were shown to have minimal phototoxicities for these chosen cell lines, which further supports the good selectivity of **GNR-Man/PRGD** and **GNR-Man** for MDA-MB-321 cells (Fig. S14†). While, no dark cytotoxicity was observed for **GNR-Man/PRGD** (Fig. S15†).

To illustrate the importance of the RGD targeting unit, PTT experiments were performed using **GNR-PEO/PRGD** and **GNR-PEO**. These GNRs lacked the mannose functionality, which enabled the targeting ability of the RGD unit to be solely evaluated. The light irradiation of MDA-MB-321 cells using **GNR-PEO/PRGD** led to the death of more cells when compared to the use of **GNR-PEO**. The light irradiation of HeLa cells using **GNR-PEO/PRGD** and **GNR-PEO** led to no differences in the number of dead cells being observed (Fig. S16†). This observation supports the targeting ability of **PRGD** on GNRs for MDA-MB-231 cells. No cytotoxicity was observed for the use of **PRGD** only (Fig. S16c†).

**Fig. 5:**
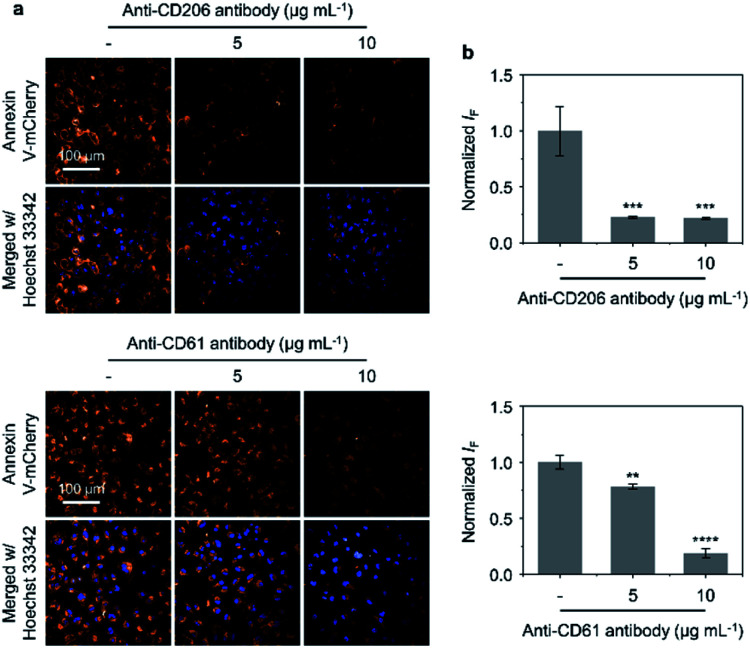
(a) Fluorescence imaging and (b) quantification of dead cells (MDA-MB-231) pre-incubated with anti-CD206 antibody (5 and 10 μg mL^−1^) (***P < 0.001 with respect to the group without antibody treatment) and anti-CD61 antibody (5 and 10 μg mL^−1^) (**P < 0.01, ****P < 0.0001 with respect to the group without antibody treatment) in the presence of **GNR-Man/PRGD** (40 μg mL^−1^/20 μM) under NIR light irradiation (808 nm, 1.0 W cm^−2^) for 3 min. Dead and total cells were stained with Annexin V-mCherry (*λ*_ex_ = 520–550 nm, *λ*_em_ = 580–650 nm) and Hoechst 33342 (*λ*_ex_ = 360–400 nm, *λ*_em_ = 410–480 nm), respectively. S. D. means standard deviation (*n* = 3).

Antibody blockade assays were subsequently carried out to evaluate the importance of the targeting units on **GNR-Man/PRGD** (mannose and **PRGD**) for their corresponding receptors (CD206 and CD61) on MDA-MB-231 cells. Annexin V-mCherry was used to visualise apoptotic cells.^54^ As shown in Fig. 5a and b, blocking receptors CD206 and CD61 with their corresponding antibodies (MRC1 and ITGB3) resulted in the reduction of the number of apoptotic cells being observed (after PTT treatment with **GNR-Man/PRGD**). This antibody-mediated reduction was shown in a concentration-dependent manner, which provides support that both MR and α_v_β_3_ receptors are targets for **GNR-Man/PRGD**. Moreover, increasing the **PRGD** concentrations on **GNR-Man** led to an increased number of apoptotic cells being observed after PTT. Finally, we noticed fewer apoptotic cells being produced when cells were incubated at 4 °C with **GNR-Man/PRGD** prior to light irradiation (Fig. S18†). This gives evidence that the cell internalization of **GNR-Man/PRGD** is kinetically controlled *via* a receptor-mediated endocytic process.^55^

**Fig. 6:**
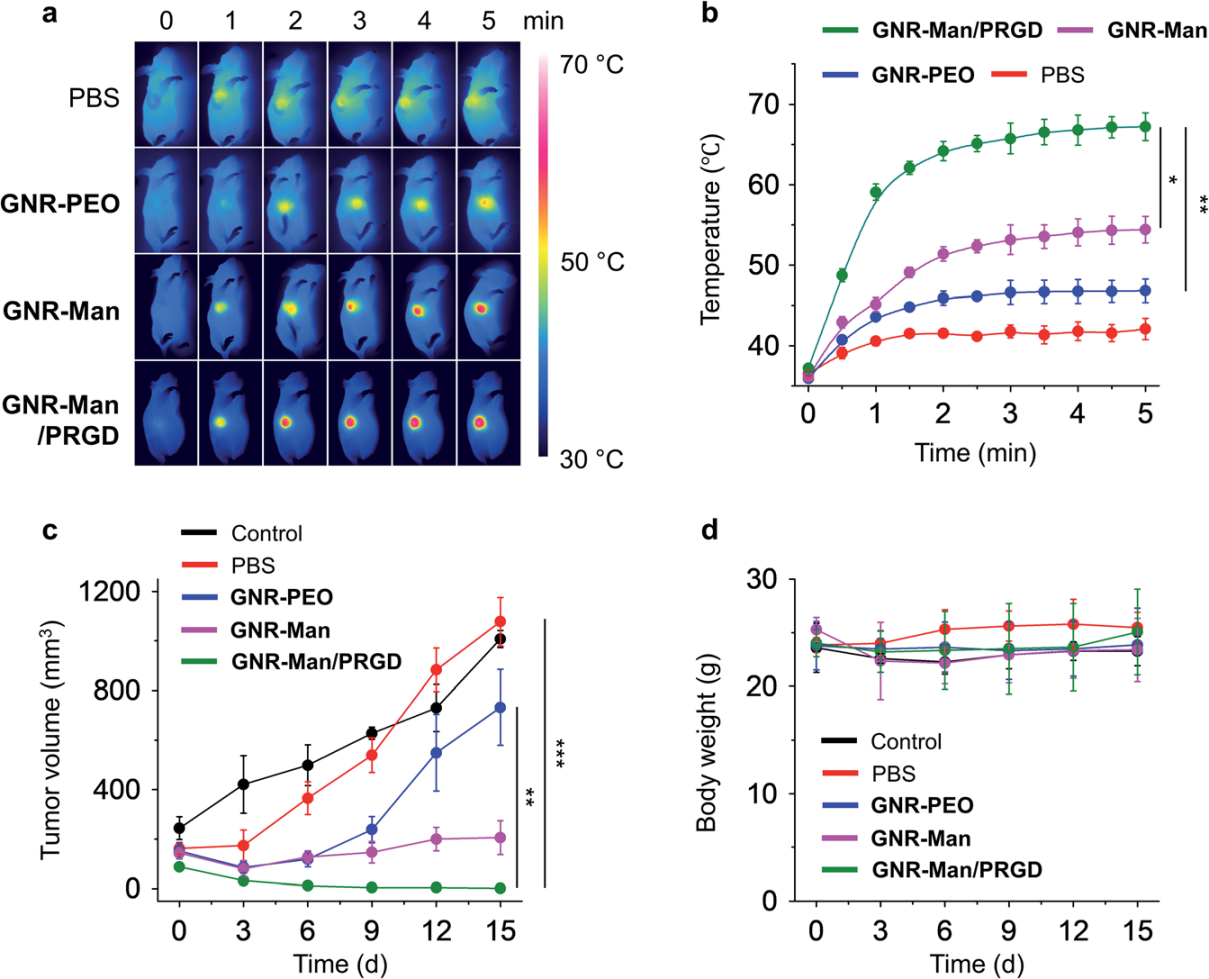
(a) Photothermal images and (b) time-dependent temperature increase at the tumor site of tumor bearing live mice (MDA-MB-231-xenograft) under 808 nm light irradiation (1.5 W cm^−2^, 5 min) (*n* = 3; for original images of all three groups, see Fig. S13†). Light irradiation was applied to the tumor site of all mice 24 h after a systemic administration of PBS (0.01 M, pH 7.40), **GNR-PEO** (0.5 mg mL^−1^), **GNR-Man** (0.5 mg mL^−1^) and **GNR-Man/PRGD** (0.5 mg mL^−1^/0.25 mM) through tail-vein injection into mice (*n* = 3); the injection volume was 5 mg kg^−1^. (c) Relative tumor volume and (d) body weight of living mice bearing MDA-MB-231-xenografts without (control) and with PTT in different groups for 15 days. *P < 0.05, **P < 0.01, ***P < 0.001. S. D. means standard deviation (*n* = 3).

**Fig. 7:**
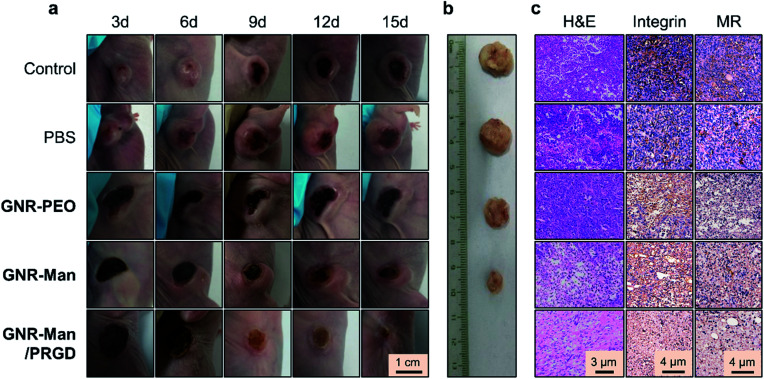
(a) Representative digital images of the tumor-bearing mice after photothermal treatment (808 nm, 1.5 W cm^−2^, 5 min) without (control) and with PBS (0.01 M, pH 7.40), **GNR-PEO** (0.5 mg mL^−1^), **GNR-Man** (0.5 mg mL^−1^) and **GNR-Man/PRGD** (0.5 mg mL^−1^/0.25 mM) for 3, 6, 9, 12 and 15 days. (b) Representative digital images of the tumors removed from the xenograft mice after PTT for 15 days. (c) H&E staining and immunohistochemical staining with integrin β3 (CD61) or MR antibody (CD206) of tumor slices removed from xenograft mice after PTT for 15 days.

With the excellent dual-receptor targeting ability of **GNR-Man/PRGD** confirmed, the PTT performance of the GNR-based agents were evaluated using tumor bearing MDA-MB-231 subcutaneous xenografts of live mice. The tumor-bearing mice (*n* = 3) were systematically administrated with PBS (0.01 M, pH 7.40), **GNR-PEO** (0.5 mg mL^−1^), **GNR-Man** (0.5 mg mL^−1^) and **GNR-Man/PRGD** (0.5 mg mL^−1^/0.25 mM) using tail-vein injection, respectively. After 24 hours post-injection, tumors were irradiated (808 nm, 1.5 W cm^−2^) for 5 min. The corresponding photothermal images were taken (Fig. 6a and S19†), and the time-dependent local temperature increase was recorded (Fig. 6b). In addition, the tumor volume (Fig. 6c) and body weight (Fig. 6d) of the mice were measured every 3 days within 15 days. The administration of each GNR did not affect the body weight of the mice (Fig. 6d). Interestingly, minimal temperature increases (42.1 °C) were observed for the control groups, PBS and **GNR-PEO**. In contrast, the local temperature at the tumor after 2 min irradiation reached 54.4 °C and 67.2 °C for **GNR-Man** and **GNR-Man/PRGD**, respectively. The greater local temperature at the tumor site for the **GNR-Man/PRGD**-treated mice suggested an enhanced accumulation of the GNRs in the tumor. Gratifyingly, for the **GNR-Man/PRGD** treatment group the volume of the tumor decreased to zero in three days, and the tumor did not regrow even after 15 days. In contrast, **GNR-Man** resulted in reduced shrinkage of tumor volume and regrowth of the tumor was observed after 6 days (Fig. 7a). It is important to note, that **GNR-Man** displayed a statistically significant therapeutic effect against the non-targeted **GNR-PEO**. This highlights the importance of mannose as a targeting unit.

To confirm the significant therapeutic efficacy of **GNR-Man/PRGD**, we removed the tumors from the mice after 15 days. No solid tumor could be isolated from the mice treated using **GNR-Man/PRGD** due to the disappearance of the tumor, whereas tumors of different sizes were obtained from the mice treated with **GNR-PEO** or **GNR-Man** (Fig. 7b). Hematoxylin-eosin (H&E) staining was performed on each isolated tumor and major organs. Typical TNBC cells were seen in the control, PBS, **GNR-PEO** and **GNR-Man** groups. In stark contrast, no TNBC cells were observed in the slice of the tissue removed from the tumor region of the mice treated with **GNR-Man/PRGD** on the fifteenth day (Fig. 7c). Subsequent immunochemical staining corroborated the absence of both integrin and MR receptors for **GNR-Man/PRGD** treated tissue slice group compared to those treated from the other groups (Fig. 7c). In addition, no obvious morphological changes were observed for the stained organ slices (Fig. S20†), suggesting the biocompatibility of **GNR-Man/PRGD**. However, before further development extensive pharmacological evaluations are required to determine the full biocompatibility of the GNRs for live subjects.

## Conclusions

In summary, we have synthesized GNR-based glycomaterials, **GNR-Gal** and **GNR-Man**, by covalently grafting glycoligands to structurally defined GNRs. **GNR-Man/PRGD** supramolecular ensembles with dual-receptor targeting function were further developed using the supramolecular self-assembly between **GNR-Man** and a pyrene-tagged RGD ligand (**PRGD**). The successful targeting of **GNR-Man/PRGD** towards both MR and α_v_β_3_ integrin receptors resulted in a highly effective PTT for TNBC *in vitro* and *in vivo*, evidenced through the nearly complete tumor ablation and no tumor regrowth being observed 15 days after PTT. These results clearly demonstrate the potential of dual-receptor targeting for effective cancer therapy. In addition, this study illustrates the potential application of structurally well-defined GNRs as photothermal agents for tumor therapy.

## Ethical statement

All animal experiments were conducted under the principles of the Regulations of Experimental Animal Administration issued by the State Committee of Science and Technology of the People's Republic of China and followed the guidelines of the Animal Care and Use Committee of Shanghai Institute of Materia Medica, Chinese Academy of Sciences.

## Data availability

All experimental data, procedures for data analysis and pertinent data including NMR of compounds used are provided in the ESI.

## Author contributions

W.-T. D. and F. X. contributed equally to this research project. J. L., Y. M. and X.-P. H. conceived the project. W.-T. D., F. X., C. X. X., J. G., H. B. R., L. Z., X. L. and J. Z. performed the experiments. G.-R. C., Y. Z., J. L., Y. M., X.-P. H. and H. T. supervised the research. W.-T. D., F. X., A. C. S., T. D. J., Y. M. and X.-P. H. wrote, and revised the paper. All the authors discussed the results and contributed to the preparation of the final manuscript.

## Conflicts of interest

There are no conflicts to declare.

## Supplementary Material

SC-012-D1SC02154K-s001
