# Supplementary material for: Graphene nanoribbon-based supramolecular ensembles with dual-receptor targeting function for targeted photothermal tumor therapy
Source: Chem Sci. 2021 Jul 15;12(33):11089–97. doi: 10.1039/d1sc02154k (PMC8386659; doi:10.1039/d1sc02154k)
Supplement: SC-012-D1SC02154K-s001 [file SC-012-D1SC02154K-s001.pdf]

Electronic Supplementary Material (ESI) for Chemical Science.
This journal is © The Royal Society of Chemistry 2021

Supporting Information

Graphene Nanoribbon-Based Supramolecular Ensembles with Dual-Receptor Targeting Function for Targeted Photothermal Tumor Therapy

Wei-Tao Dou,[a]‡ Fugui Xu,[b]‡ Chen-Xi Xu,[a,c] Jie Gao,[a,c] Hong-Bo Ru,[c] Xiangfeng Luan,[b] Jiacheng Zhang,[b] Ling Zhu, [a,c] Adam C. Sedgwick,[d] Guo-Rong Chen,[a] Yi Zang,[c] Tony D. James,[e,f] He Tian,[a] Jia Li,*[c] Yiyong Mai,*[b] and Xiao-Peng He*[a]

[a]*Key Laboratory for Advanced Materials and Joint International Research Laboratory of Precision Chemistry and Molecular Engineering, Feringa Nobel Prize Scientist Joint Research Center, School of Chemistry and Molecular Engineering, Frontiers Center for Materiobiology and Dynamic Chemistry, East China University of Science and Technology, 130 Meilong RD, Shanghai 200237, P. R. China. E-mail: xphe@ecust.edu.cn*

[b]*School of Chemistry and Chemical Engineering, Frontiers Science Center for Transformative Molecules, Shanghai Key Laboratory of Electrical Insulation and Thermal Ageing, Shanghai Jiao Tong University, 800 Dongchuan RD, Shanghai 200240, P. R. China. E-mail: mai@sjtu.edu.cn*

[c]*National Center for Drug Screening, State Key Laboratory of Drug Research, Shanghai Institute of Materia Medica, Chinese Academy of Sciences, 189 Guo Shoujing Rd., Shanghai 201203, P. R. China. E-mail: jli@simm.ac.cn*

[d]*Department of Chemistry, The University of Texas at Austin, Austin, Texas, 78712-1224, United States*

[e]*Department of Chemistry, University of Bath, Bath, BA2 7AY, UK.*

[f]*School of Chemistry and Chemical Engineering, Henan Normal University, Xinxiang 453007, P. R. China.*

‡*These authors contributed equally*

Table of Content

1. **Experimental section**··········S3-S9
2. **Additional figures**··········S10-S22
3. **Spectra of new compounds**··········S23-S27
4. **Additional references**··········S28

1. Experimental sections

General information.

All purchased chemicals and reagents are of analytical grade. Transmission electron microscopic (TEM) images were obtained using a JEOL 2100. Dynamic light scattering (DLS) and Zeta potential were carried out using a LB-550 DLS Nano-Analyzer (Horiba, Japan). AFM images were recorded using a scanning probe microscope (Multimode Nanoscope, USA) operated in tapping mode using silicon nitride cantilevers with a force constant of 0.12 N m^{-1}. Fourier transform infrared (FTIR) spectra were recorded on a Spectrum 100 (Perkin Elmer, Inc., USA) spectrometer. One hundred scans were collected for each sample at a resolution of 4 cm^{-1} over the wavenumber region 4000–400 cm^{-1}. Samples were prepared in KBr discs. Thermal gravimetric analysis (TGA) was measured using a TA-Instruments Q5000IR thermo gravimetric analyzer in flowing (100 mL min^{-1}) nitrogen with an increasing temperature rate of 10 °C min^{-1}. UV-vis absorption spectra were recorded on a Varian Cary 100 Spectrophotometer. ^{1}H and ^{13}C NMR spectra were recorded on a Bruker AM-400 spectrometer. Raman spectra were recorded with a Renishaw in Via-Reflex laser Micro-Raman spectrometer and excited by an argon ion laser (*I* = 532 nm). Photothermal conversion data were recorded with a Nikon thermal imaging camera and 808 nm infrared laser (Changchun New Industries Optoelectronics Tech. Co., Ltd., China). MTS assays were carried out using a M5 microplate reader (Molecular Device, USA) and the fluorescence images were recorded using an Operetta high content imaging system and quantified by the Columbus image data analysis system (PerkinElmer, US).

Synthesis of Alkynyl-Gal and Alkynyl-Man.

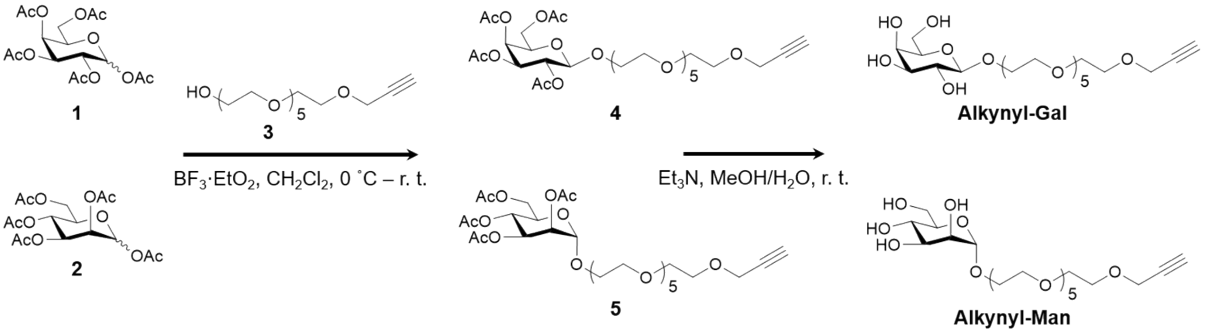

Scheme S1. Synthetic route of **Alkynyl-Gal** and **Alkynyl-Man**

Compound **3** (3,6,9,12,15,18-Hexaoxaheneicos-20-yn-1-ol) was synthesized according to reported procedures.[1]

Synthesis of compound 4 and 5. 3, 6, 9, 12, 15, 18-Hexaoxaheneicos-20-yn-1-ol (300 mg, 0.94 mmol) and D-galactopyranose or D-gannopyranose (366 mg, 0.94 mmol) were added into 20 mL anhydrous dichloromethane. After the mixture was cooled to 0 ℃, dry boron trifluoride etherate (0.12 mL, 0.94 mmol) was added dropwise under N_2. The resulting mixture was stirred

at room temperature for 24 h. Then, the mixture was washed with saturated brine, and the organic layer was concentrated under vacuum to give a crude product, which was purified by column chromatography (CH_2Cl_2/MeOH, 15/1, v/v) to afford compound **4** or compound **5** as a white solid.

Compound **4** (494 mg, 81%). ^{1}H NMR (400 MHz, $CDCl_3$) δ 5.32 (d, J = 3.0 Hz, 1H, *H*-3), 5.25 (br, s, 1H, *H*-5), 5.16-5.09 (m, 1H, *H*-2), 4.95 (dd, J = 10.5, 3.4 Hz, 1H, *H*-1), 4.55–4.46 (m, 1H, *H*-4), 4.14 (br, s, 2H, *H*-6a, *H*-6b), 3.91–3.84 (m, 2H, CH_2C≡CH), 3.59 (m, 24H, 6-OCH_2CH_2O), 2.41–2.40 (m, 1H, CH_2C≡C*H*), 2.08 (s, 3H, COCH_3), 1.99 (s, 3H, COCH_3), 1.98 (s, 3H, COCH_3), 1.92 (s, 3H, COCH_3); ^{13}C NMR (101 MHz, $CDCl_3$) δ 170.4, 170.3, 170.1, 169.5, 101.3, 79.6, 74.7, 74.6, 70.9, 70.6, 70.5, 70.4, 70.2, 69.1, 68.8, 67.0, 67.0, 63.6, 61.3, 20.8, 20.7, 20.6. HR-ESI-MS (*m/z*): $[M+K]^+$ calcd for $C_{29}H_{46}O_{16}K$, 689.2423, found 689.2418.

Compound **5** (420 mg, 69%). ^{1}H NMR (400 MHz, $CDCl_3$) δ 5.36 (dd, J = 10.1, 3.4 Hz, 1H, *H*-3), 5.27 (dd, J = 3.3, 1.8 Hz, 1H, *H*-1), 4.97–4.82 (m, 1H, *H*-2), 4.30 (dd, J = 12.4, 5.1 Hz, 1H, *H*-4), 4.29–4.28 (m, 3H, CH_2C≡CH, *H*-5), 4.13–4.04 (m, 2H, *H*-6a, *H*-6b), 3.87–3.78 (m, 1H, CH_2C≡C*H*), 3.67–3.66 (m, 24H, 6-OCH_2CH_2O), 2.16 (s, 3H, COCH_3), 2.11 (s, 3H, COCH_3), 2.05 (s, 3H, COCH_3), 1.99 (s, 3H, COCH_3); ^{13}C NMR (101 MHz, $CDCl_3$) δ 170.7, 170.0, 169.9, 169.7, 97.7, 79.6, 74.6, 74.6, 70.7, 70.5, 70.4, 69.9, 69.5, 69.1, 68.3, 67.3, 66.1, 62.4, 58.4, 20.9, 20.8, 20.7. HR-ESI-MS (*m/z*): $[M+K]^+$ calcd for $C_{29}H_{46}O_{16}K$, 689.2423, found 689.2421.

Synthesis of Alkynyl-Gal and Alkynyl-Man. Compound **4** or Compound **5** (200 mg, 0.31 mmol) and sodium methoxide (70 mg, 1.24 mmol) were dissolved in 20 mL anhydrous methanol. The resulting mixture was stirred overnight at room temperature. The solvent was removed under reduce pressure, and the residue was directly purified by column chromatography on silica gel (CH_2Cl_2/MeOH = 5:1, v/v) to afford **Alkynyl-Gal** or **Alkynyl-Man** as a colourless liquid.

Alkynyl-Gal (134 mg, 90%). ^{1}H NMR (400 MHz, CD_3OD) δ 4.27 (d, J = 8.4 Hz, 1H, *H*-1), 4.20 (d, J = 2.4 Hz, 2H, CH_2C≡CH), 4.06–3.99 (m, 1H, *H*-3), 3.83 (d, J = 2.6 Hz, 1H, *H*-2), 3.78–3.75 (m, 1H, *H*-5), 3.74–3.62 (m, 24H, 6-OCH_2CH_2O), 3.54 (dd, J = 6.8, 4.5 Hz, 1H, *H*-4), 3.52–3.45 (m, 2H, *H*-6a, *H*-6b), 2.88 (t, J = 2.4 Hz, 1H, CH_2C≡C*H*); ^{13}C NMR (101 MHz, DMSO-d_6) δ 103.5, 80.3, 77.1, 77.1, 75.1, 73.4, 70.4, 69.7, 69.5, 68.5, 68.0, 67.7, 62.8, 60.3, 57.5. HR-ESI-MS (*m/z*): $[M+Na]^+$ calcd for $C_{21}H_{38}O_{12}Na$, 505.2261, found 505.2248.

Alkynyl-Man (137 mg, 92%). ^{1}H NMR (400 MHz, DMSO-d_6) δ 4.78-4.68 (m, 2H, *H*-1, *H*-3), 4.62 (dd, J = 8.1, 3.5 Hz, 2H, *H*-2, *H*-4), 4.46 (t, J = 6.0 Hz, 1H, *H*-5), 4.14 (d, J = 2.4 Hz, 2H, CH_2C≡CH), 3.71–3.61 (m, 2H, *H*-6a, *H*-6b), 3.56–3.41 (m, 24H, 6-OCH_2CH_2O), 3.33–3.27 (m, 1H, CH_2C≡C*H*); ^{13}C NMR (101 MHz, DMSO-d_6) δ 99.9, 80.3, 77.1, 77.1, 73.9, 70.9, 70.2, 69.7, 69.5, 68.5, 66.9, 65.7, 62.7, 61.2, 57.4. HR-ESI-MS (*m/z*): $[M+Na]^+$ calcd for

$C_{21}H_{38}O_{12}Na$, 505.2261, found 505.2245.

Synthesis of GNR-Gal and GNR-Man.

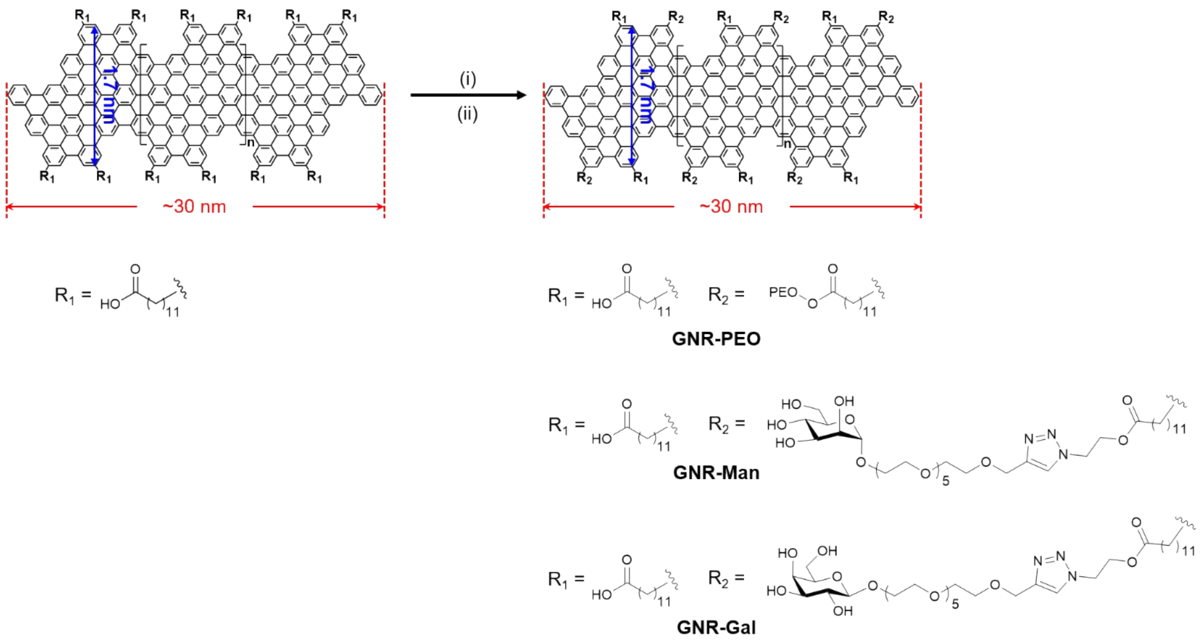

Scheme S2. Synthetic route of **GNR-Gal and GNR-Man**. Reagents and conditions: i) DCC (N,N'-dicyclohexylcarbodiimide), DMAP (4-dimethylamino-pyridine) and $HOCH_2CH_2N_3$ in THF; ii) CuBr, Me_6TREN (tris[2-(dimethylamino)ethyl]amine), **Alkynyl-Gal**/ **Alkynyl-Man** in THF.

GNR-COOH and **GNR-PEO** were synthesized according to previously reported procedures.[2]

Synthesis of GNR-N_3. To a flame-dried reaction flask, **GNR-COOH** (20.00 mg, 0.24 mmol) and 40 mL anhydrous THF were added. The resulting mixture was ultrasonically dispersed for 4 h to obtain a blue-black solution. Subsequently, 2-azido-ethanol (52.20 mg, 0.60 mmol), DMAP (0.30 mg, 24.0 μmol) and DCC (50.00 mg, 0.24 mmol) were added to the solution, and cooled with ice water bath for 30 min. The ice bath was removed, and the mixture was stirred for 3 days at room temperature, and then sonicated for 0.5 h every 2 h. THF was removed under reduced pressure, and then DMF was added for filtration. The filter cake was sequentially washed with DMF and CH_2Cl_2, and finally vacuum dried to obtain **GNR-N_3**.

Synthesis of GNR-Gal and GNR-Man. **Alkynyl-Gal** or **Alkynyl-Man** (10 mg, 0.12 mmol) was added to the mixed solution of **GNR-N_3** and THF (20 mL), and the mixture was bubbled with nitrogen for 30 min. Then, 2 mL of a THF solution containing CuBr (1.43 mg, 0.01 mmol) and tris (2-dimethylaminoethyl) amine (2.30 mg, 0.01 mmol) was added to the mixture, and bubbled with nitrogen for 15 min. The reaction was placed in an oil bath at 60 ℃ for 72 h, and then sonicated for 0.5 h every 2 h. THF was removed in vacuum, and then DMF was added for

filtration. The filter cake was sequentially washed with DMF and CH_2Cl_2, and finally vacuum dried to obtain **GNR-Gal** or **GNR-Man**.

Peptide ligand. PRGD (Pyrenyl-KKKRGD) was purchased from Ziyu Biotechnology Co., Ltd. (Shanghai, China). The purified peptide was analyzed by RP-HPLC (C-18) using 30% solvent A (0.1% TFA in acetonitrile) and 70% solvent B (0.1% TFA in water) to demonstrate a purity of 96.7%. LCMS (MALDI-TOF): $[M+2H]^{2+}$ calcd for 501.6, found 501.4.

The preparation of GNR-Man/PRGD. The supramolecular self-assembly of **GNR-Man/PRGD** was achieved by mixing **GNR-Man** (40 μg mL^{-1}) with **PRGD** (20 μM) in a PBS (0.01 M, pH 7.40, 0.5 % Triton X-100 (TX-100), v/v) solution, followed by sonication for 15 min. The resulting solution was stored under refrigeration at 4 ^{o}C and used as-prepared.

Transmission electron microscopy (TEM). GNRs (50 μg mL^{-1}) or **GNR-Man/PRGD** (10 μg mL^{-1}/5 μM) were dropped onto 300 mesh holey carbon copper grids, followed by air-drying at room temperature, for TEM characterizations. JEOL 2100 equipped with a Gatan Orius charged-coupled device camera and Tridiem energy filter operating at 200 kV was used for HRTEM, and data were processed using Image J software.

Atomic force microscopy (AFM). GNRs (100 μL, 2.5 μg mL^{-1}) were dropped onto a freshly cleaved mica surface, followed by drying at room temperature. The AFM images were then recorded with a scanning probe microscope (Multimode Nanoscope, USA) operated in tapping mode using silicon nitride cantilevers with a force constant of 0.12 N m^{-1}.

Photothermal measurement. A system containing 200 μL of GNRs with different concentrations (10, 20, 30, 40, 50 μg mL^{-1}) was exposed to NIR light irradiation (808 nm, 1 W cm^{-2}) for 2 min. Then, the photothermal images were recorded with a thermal imaging equipment (Nikon) and quantified by the analysis program ThermoX. The photo-conversion efficiency (PCE, η) of GNRs was calculated *via* equation (1).[3]

$$\eta = \frac{hS(T_{max} - T_{surr}) - Q_{dis}}{I(1 - 10^{-A_{\lambda}})} \quad (1)$$

where h is the heat transfer coefficient, S is the surface area of the container, T_{max} and T_{surr} are the maximum steady-state temperature and the surrounding temperature of the environment, respectively, Q_{dis} is the heat dissipation from the light absorbed by solvent and container, I is the incident laser power, and A_{λ} is the absorbance intensity of the sample at 808 nm. hS is determined *via* Equation (2).

$$\tau_s = \frac{m_D c_D}{hS} \tag{2}$$

where m_D and c_D are the mass and heat capacity (4.2 J g^{-1}) of the deionized water, respectively. To obtain the value of τ_s, we first determined the value of θ, which is defined as the ratio of ΔT to ΔT_{max}, via equation (3). Then, the value of τ_s was derived *via* Equation (4).

$$\theta = \frac{\Delta T}{\Delta T_{max}} = \frac{T - T_{max}}{T_{max} - T_{surr}} \tag{3}$$

$$t = -\tau_s \ln\theta + b \tag{4}$$

where τ_s is the time constant for heat transfer of the system, which was determined from Figs. 2b, 2f and 2j in the main text. Q_{dis} is calculated *via* Equation (5).

$$Q_{dis} = \frac{m_D c_D\,(T_{max(water)} - T_{surr})}{\tau_{water}} \tag{5}$$

Cell viability. Cells were plated overnight on 96-well plates at a density of 8000 cells $well^{-1}$ in growth medium. After seeding, cells were treated with GNRs at different concentrations for 15 min (n = 3). Then, cells were gently washed with PBS once. After exposure for 48 h, the MTS/PMS (20:1, Promega Corp) solution (10 μL $well^{-1}$) was added to each well containing 100 μL of serum-free HG-DMEM, followed by gently shaking. After incubation at 37 °C under 5% CO_2 for 2 h, 80 μL of GNRs was transferred to another 96-well plate. The absorbance of the mixture solutions was measured at 490 nm with that at 650 nm as a reference using an M5 microplate reader (Molecular Device, USA). The optical density of the result in MTS assay was directly proportional to the number of viable cells.

Cell culture. Hep-G2 and MDA-MB-231 cells were maintained in a Gibco™ RPMI 1640 Medium (Invitrogen, Carlsbad, CA, USA) supplemented with 5% fetal bovine serum (Gibco, Gland Island, NY, USA). Cells were cultured in a humidified atmosphere of 5% CO_2 and 95% air at 37℃ and split when reached 90% confluency.

Fluorescence imaging of cells. MDA-MB-231 and Hep-G2 cells (2.5×10^4 $well^{-1}$) were seeded onto a black 96-well microplate with optically clear bottom (Greiner bio-one, Germany) overnight. Then, the cells were incubated with GNRs at final concentrations of 0, 10, 20 and 40 μg mL^{-1} (diluted with PBS) for 5 min (n = 3). The cells were continuously irradiated by laser for 3 min with different laser powers (0, 1 and 2 W cm^{-2}), and then were gently washed with PBS. Subsequently, the cells were incubated with a mixture of SYTOXGreen (1:2500 diluted by PBS)/Annexin V-mCherry (1:100 diluted by PBS) and Hoechst (1:3000 diluted by

PBS) for 10 min, and then washed by PBS. The fluorescence images were recorded using an Operetta high content imaging system and quantified by the Columbus image data analysis system (Perkinelmer, US). Fluorescence imaging and quantification of dead MDA-MB-231 and HeLa cells that were incubated with **GNR-PEO** (40 μg mL^{-1}) with and without **PRGD** (20 μM) or **GNR-Man** with increasing concentrations fo **PRGD** (0-40 μM) were carried out in a similar way (n=3).

Blockade assay experimentation. MDA-MB-231 cells (2.5×10^4 $well^{-1}$) were seeded onto a black 96-well microplate with optically clear bottom (Greiner bio-one, Germany) overnight. After 24 h, cells (n = 3) were incubated with MR-specific (CD206, MRC1) or integrin $\alpha_v\beta_3$-specific (CD61, ITGB3) antibody (5 and 10 μg mL^{-1}) at 4 ℃ for 1.5 h (n=3). Then, **GNR-Man/PRGD** (40 μg mL^{-1}/20 μM) was added, incubated at 37 ℃ for 30 min, and then irradiated by a NIR laser (808 nm, 1 W cm^{-2}) for 3 min. The cells were washed with PBS three times. Then, dead and total cells were stained with Annexin V-mCherry (λ_{ex} = 520-550 nm, λ_{em} = 580-650 nm) and Hoechst 33342 (λ_{ex} = 360-400 nm, λ_{em} = 410-480 nm), respectively.

Incubation temperature assay. Cells were separately plated on two clear-bottom 96-well plates (Greiner bioone, Germany) in growth medium overnight. The cells were pre-incubated at different temperature points (4 ℃ and 37 ℃) for 1 h. Then, cells were treated with **GNR-Man/PRGD** (40 μg mL^{-1}/20 μM) for 20 mins at 37 ℃ under 5% CO_2 (n = 3). Subsequently, cells were gently washed with PBS three times, and then irradiated by 808 nm light for 3 min with a laser power of 1 W cm^{-2}. Then, cells were stained with a mixture of Annexin V-mCherry (1:100 diluted by PBS) and Hoechst 33342 (1:2000 diluted by PBS) for 20 min, and then washed by PBS. The fluorescence images were recorded using an Operetta high content imaging system and quantified by the Columbus image data analysis system (Perkinelmer, US) with Annexin V-mCherry (λ_{ex} = 520-550 nm, λ_{em} = 580-650 nm) and Hoechst 33342 (λ_{ex} = 360-400 nm, λ_{em} = 410-480 nm), respectively.

Photothermal imaging of xenograft-bearing mice models *in vivo*. All animal experiments were conducted under the principles of the Regulations of Experimental Animal Administration issued by the State Committee of Science and Technology of the People's Republic of China and followed the guidelines of the Animal Care and Use Committee of Shanghai Institute of Materia Medica, Chinese Academy of Sciences.

Female 4−6-week-old Nu/Nu nude mice were purchased from Beijing Vital River Laboratory Animal Technology Co. Ltd. The MDA-MB-231 subcutaneous xenograft models were established by injecting the right armpit of Nu/Nu nude mice with 100 μL of MDA-MB-231 cells at a cell density of 5×10^6. A tumor volume of up to 300 mm^{-3} was considered to be amenable for photothermal imaging experiments *in vivo*. Different solutions including PBS (0.01 M, pH 7.40), **GNR-PEO** (0.5 mg mL^{-1}), **GNR-Man** (0.5 mg mL^{-1}) and **GNR-Man/PRGD** (0.5 mg mL^{-1}/0.25 mM) were injected into the xenograft mice through tail-vein

into mice (n = 3), where the injection volume was 5 mg Kg^{-1}. After 24 h post-injection, the mice were anesthetized with Zoletil (0.05 mL/10 g) through intraperitoneal injection, and the photothermal images and the dynamic temperature increase at the tumor site were recorded with thermal imaging equipment (Nikon) under 808 nm light (1.5 W cm^{-2}) irradiation for 5 min. The tumor volume and body weight of mice were measured every 3 days for 15 days. The tumor volume was calculated by $V = (A \times B^2)/2$, where A and B are the longest and shortest diameter of tumor, respectively.[4] After 15 days, mice were sacrificed to obtain paraffin sections of tumor and differernt organs for H&E and immunochemical staining analyses.

2. Additional Figures

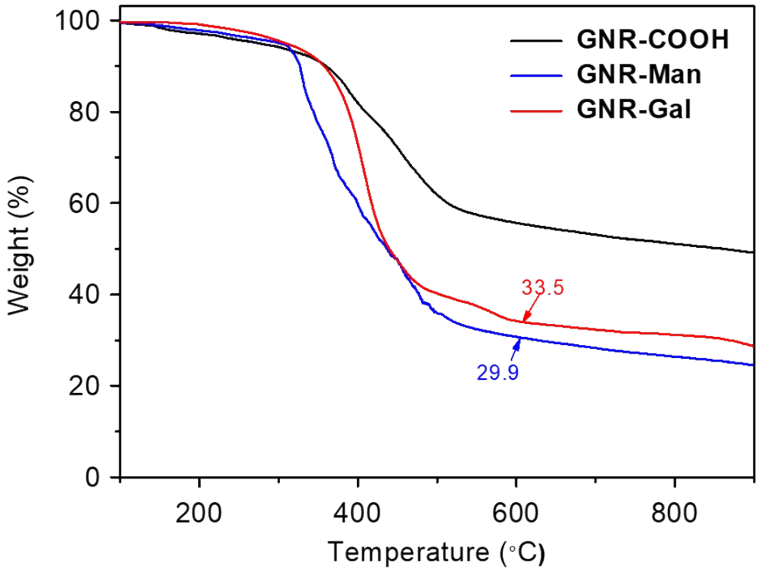

Figure S1. TGA curves of **GNR-COOH**, **GNR-Gal** and **GNR-Man** under N_2 (100 mL min^{-1}) with an increasing temperature rate of 10 °C min^{-1}.

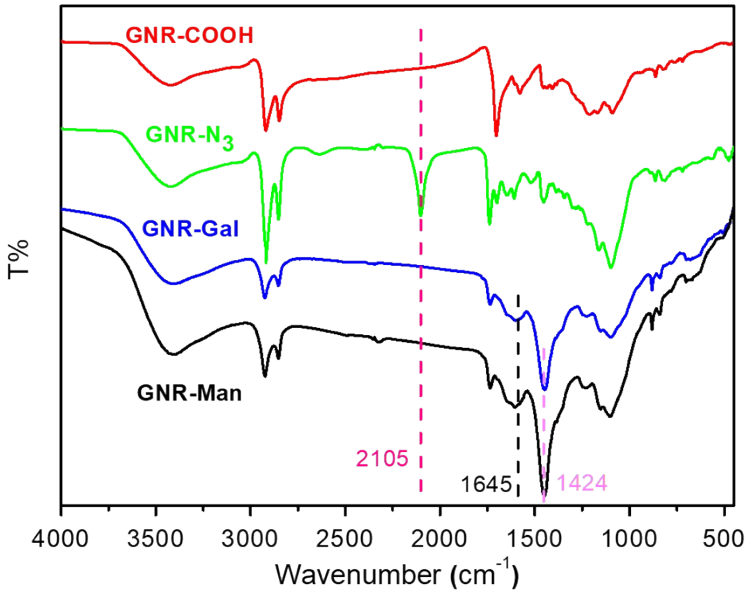

Figure S2. FTIR spectra of **GNR-COOH**, **GNR-N$_3$**, **GNR-Gal** and **GNR-Man**. One hundred scans were collected for each sample at a resolution of 4 cm^{-1} over the wavenumber region, 4000–400 cm^{-1}. Samples were prepared in KBr discs. The emergence of new absorption bands assigned to carbohydrate ligands (ν_{O-H} = 1645 cm^{-1} and $\nu_{C=C}$ = 1424 cm^{-1}) and the decrease in **GNR-N$_3$**'s absorption band at ν_{N_3} = 2105 cm^{-1} for **GNR-Gal** and **GNR-Man** suggest the successful grafting of O-alkynyl-galactoside/O-alkynyl-mannoside onto GNRs.

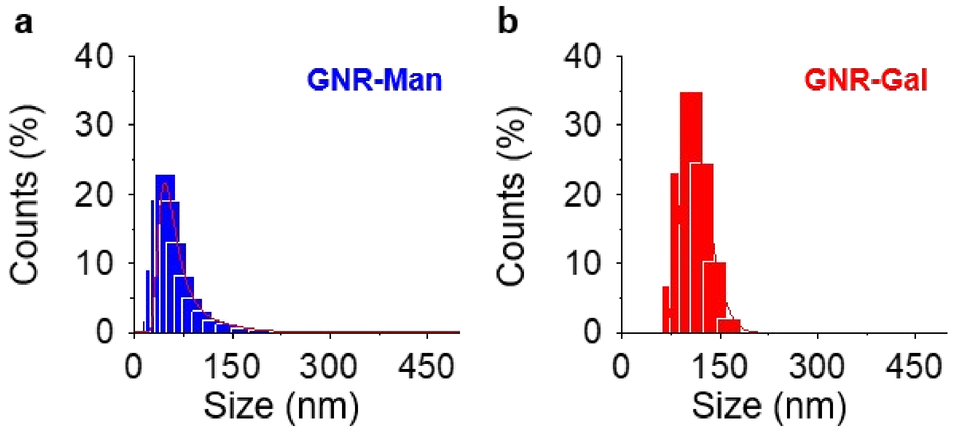

Figure S3. Dynamic light scattering analyses of (a) **GNR-Man** and (b) **GNR-Gal** in PBS (0.01 M, pH 7.40, 0.5% TX-100, v/v).

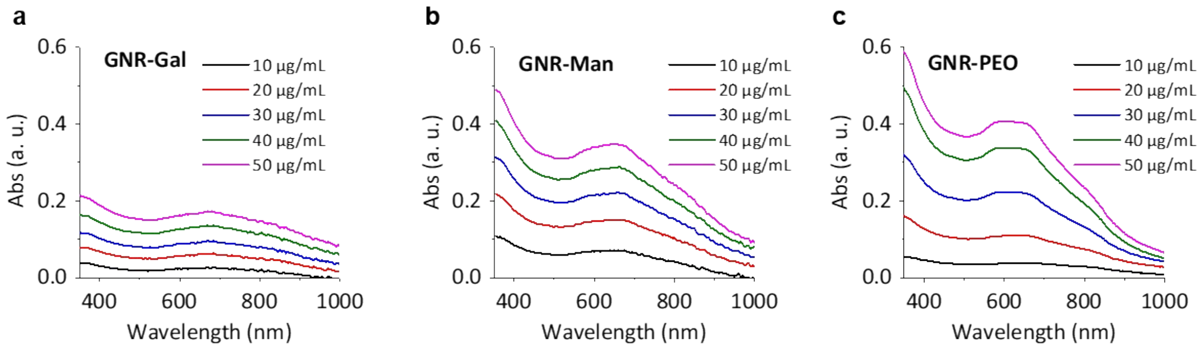

Figure S4. UV-vis absorption spectra of (a) **GNR-Gal**, (b) **GNR-Man** and (c) **GNR-PEO** at different indicated concentrations in PBS (0.01 M, pH 7.40, 0.5 % TX-100, v/v).

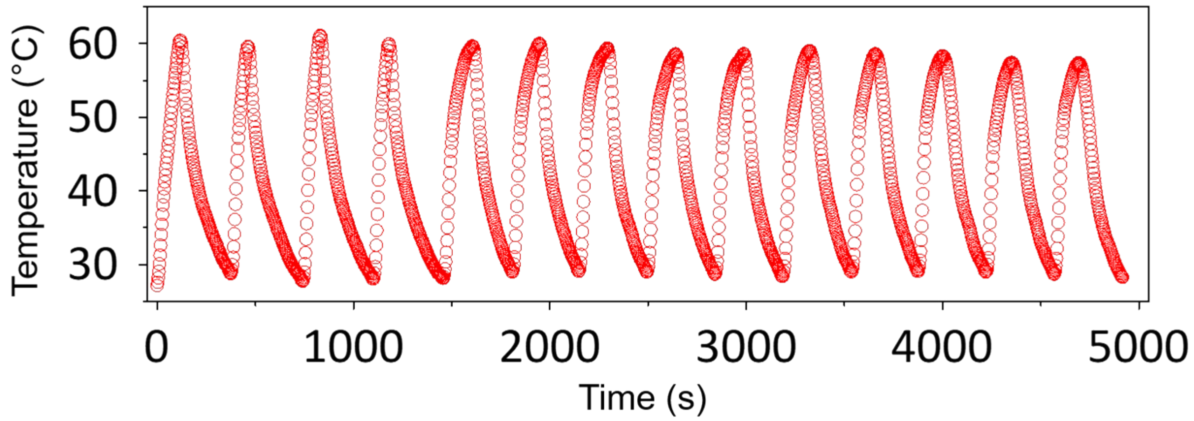

Figure S5. Heating (808 nm, 1.0 W cm^{-2}) and cooling cycles of **GNR-Man** (40 μg mL^{-1}) measured in PBS (0.01M, pH 7.40, 0.5 % TX-100, v/v).

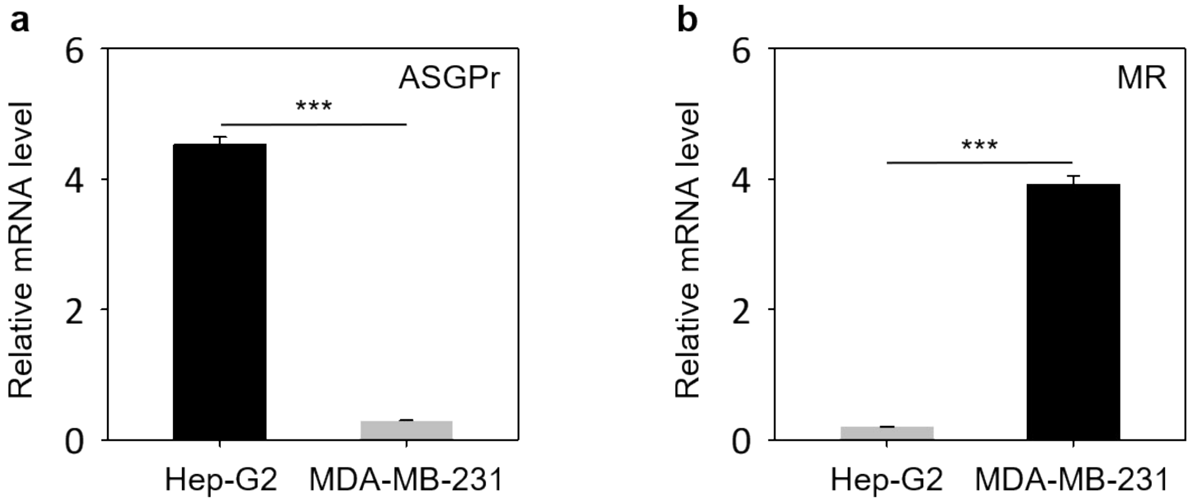

Figure S6. Relative mRNA level of (a) ASGPr and (b) MR in Hep-G2 and MDA-MB-231 cells determined by real-time quantitative polymerase chain reaction (***$P < 0.001$).

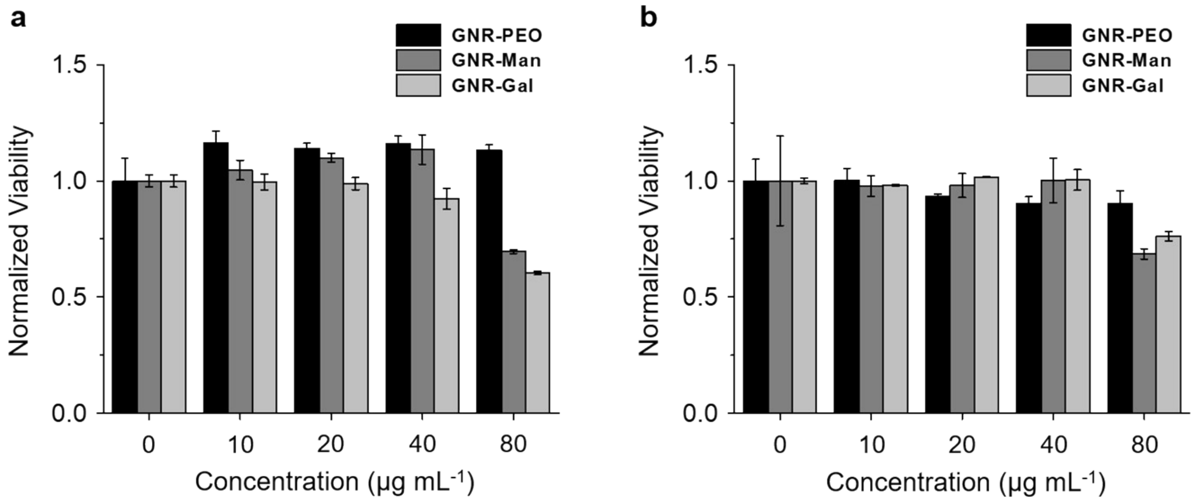

Figure S7. Cell viability treated with aqueous dispersions of the GNRs measured by an MTS assay. (a) Hep-G2 cells incubated with increasing GNR concentrations (0, 10, 20, 40 and 80 μg mL^{-1}), and (b) MDA-MB-231 cells incubated with increasing GNR concentrations (0, 10, 20, 40 and 80 μg mL^{-1}). The GNRs were incubated with the cells for 30 min prior to a 72 hours cell viability assay.

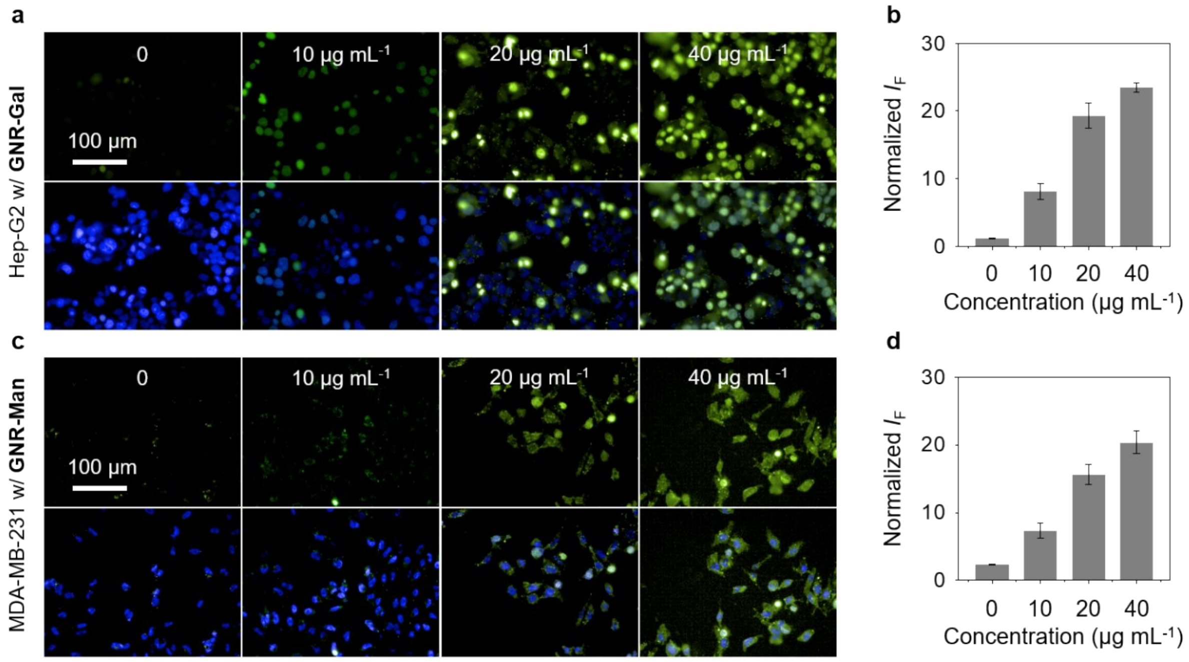

Figure S8. (a) Fluorescence imaging and (b) quantification of dead cells of Hep-G2 incubated with increasing **GNR-Gal** concentration (0, 10, 20 and 40 μg mL^{-1}) under NIR light irradiation (808 nm, 1.0 W cm^{-2}) for 3 min. (c) Fluorescence images and (d) quantification of dead cells of MDA-MB-231 incubated with increasing **GNR-Man** concentration (0, 10, 20 and 40 μg mL^{-1}) under NIR light irradiation (808 nm, 1.0 W cm^{-2}) for 3 min. Scale bar is applicable to all images. The dead and total cells were stained with SYTOX Green (λ_{ex} = 490-510 nm, λ_{em} = 530-590 nm) and Hoechst 33342 (λ_{ex} = 360-400 nm, λ_{em} = 410-480 nm), respectively.

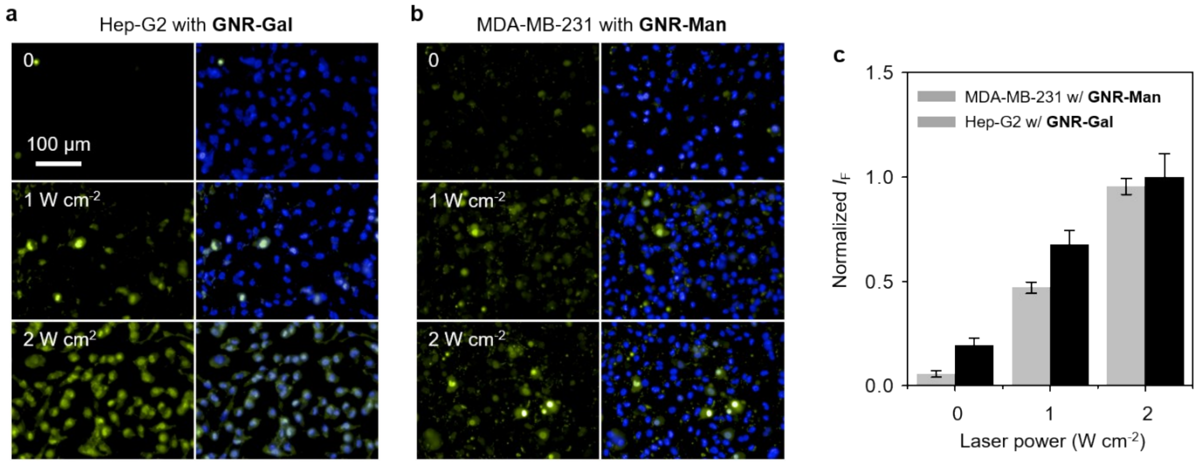

Figure S9. (a) Fluorescence imaging and quantification (c) of dead cells of Hep-G2 incubated with **GNR-Gal** (40 μg mL^{-1}) under NIR light irradiation (808 nm; 0, 1 and 2 W cm^{-2}) for 3 min. Fluorescence imaging (b) and quantification (c) of dead cells of MDA-MB-231 incubated with **GNR-Man** (40 μg mL^{-1}) under NIR light irradiation (808 nm; 0, 1 and 2 W cm^{-2}) for 3 min. Scale bar is applicable to all the images. The dead and total cells were stained with SYTOX Green (λ_{ex} = 490-510 nm, λ_{em} = 530-590 nm) and Hoechst 33342 (λ_{ex} = 360-400 nm, λ_{em} = 410-480 nm), respectively.

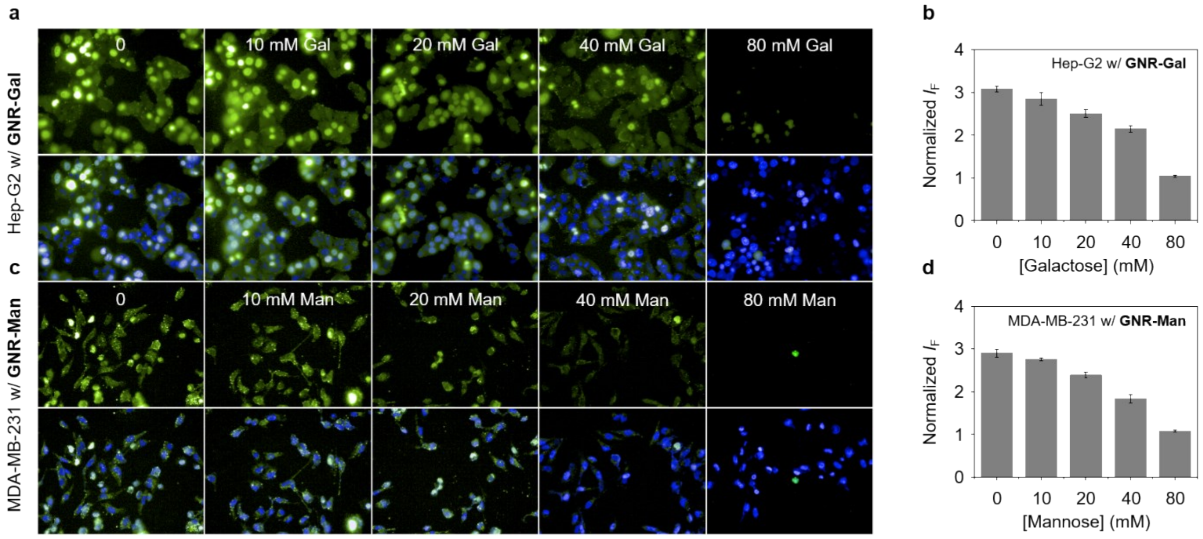

Figure S10. (a) Fluorescence imaging (b) quantification of dead cells of Hep-G2 incubated with **GNR-Gal** (40 μg mL^{-1}) in the presence of increasing free galactose under NIR light irradiation (808 nm, 1.0 W cm^{-2}) for 3 min. (c) Fluorescence imaging and (d) quantification of dead cells of MDA-MB-231 incubated with **GNR-Man** (40 μg mL^{-1}) in the presence of increasing free mannose under NIR light irradiation (808 nm, 1.0 W cm^{-2}) for 3 min. Scale bar is applicable to all the images. The dead and total cells were stained with SYTOX Green (λ_{ex} = 490-510 nm, λ_{em} = 530-590 nm) and Hoechst 33342 (λ_{ex} = 360-400 nm, λ_{em} = 410-480 nm), respectively.

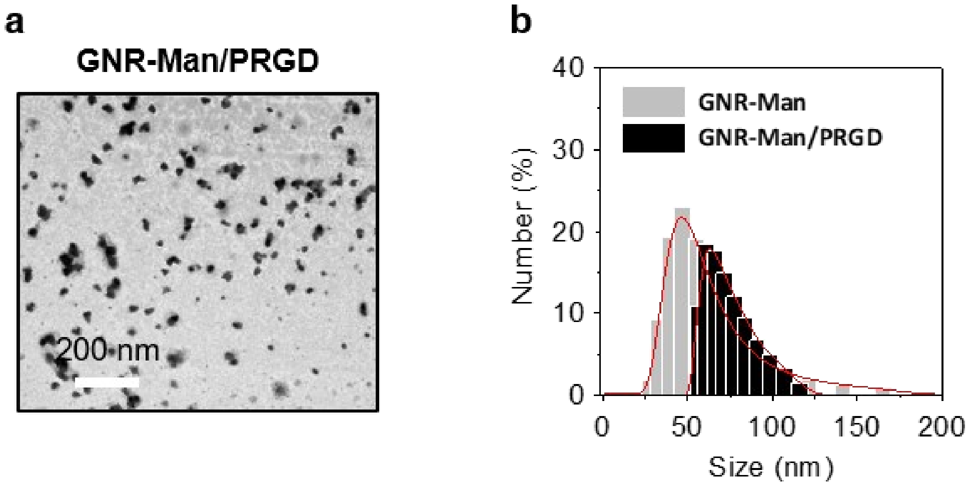

Figure S11. (a) Representative TEM image of the superstructures formed by **GNR-Man/PRGD** (10 μg mL^{-1}/5 μM) in water (0.5 % TX-100, v/v). (b) Dynamic light scatting analyses of **GNR-Man/PRGD** (10 μg mL^{-1}/5 μM) in PBS (0.01 M, pH 7.40, 0.5 % TX-100, v/v).

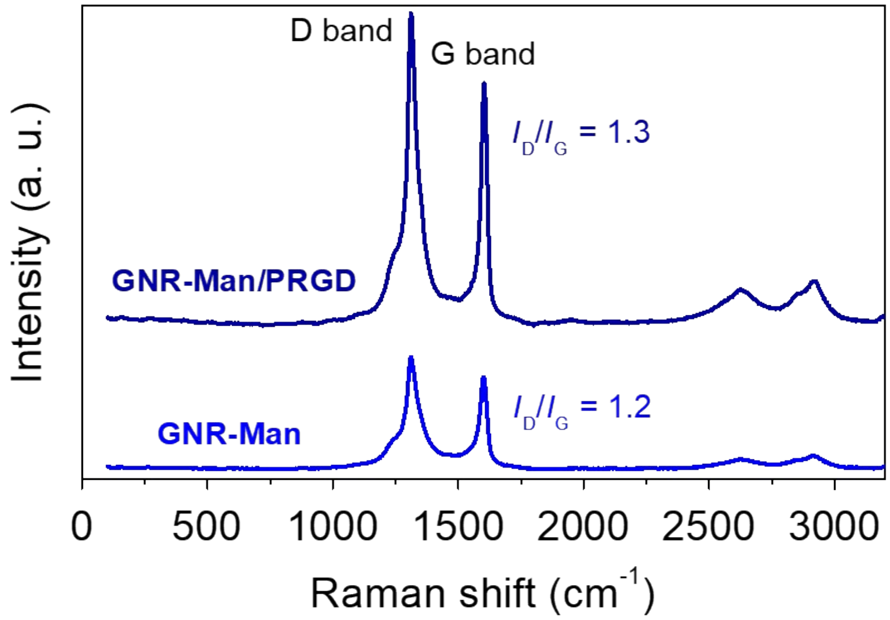

Figure S12. Raman spectra of **GNR-Man** (40 μg mL^{-1}) with and without **PRGD** (20 μM) excited by an argon ion laser (I = 532 nm).

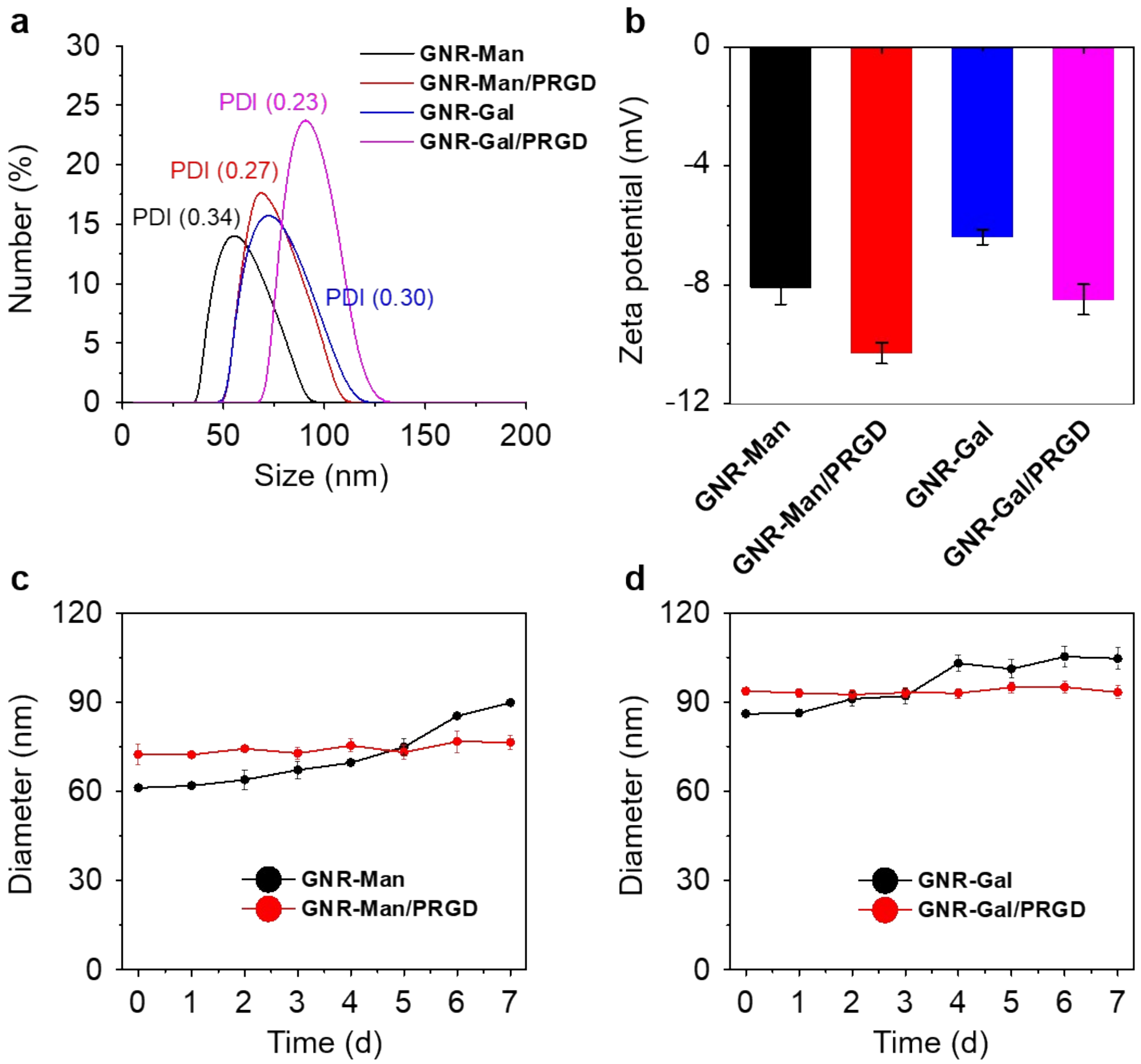

Figure S13. (a) Dynamic light scattering and (b) zeta potential of **GNR-Man** (40 μg mL^{-1}), **GNR-Man/PRGD** (40 μg mL^{-1}/20 μM), **GNR-Gal** (40 μg mL^{-1}) and **GNR-Gal/PRGD** (40 μg mL^{-1}/20 μM) in PBS (0.01 M, pH 7.40, 0.5% TX-100, v/v). Plotting changes in hydrodynamic diameter of (c) **GNR-Man** (40 μg mL^{-1}) and **GNR-Man/PRGD** (40 μg mL^{-1}/20 μM), and (d) **GNR-Gal** (40 μg mL^{-1}) and **GNR-Gal/PRGD** (40 μg mL^{-1}/20 μM) over the course of time in PBS (0.01 M, pH 7.40, 0.5% TX-100, v/v).

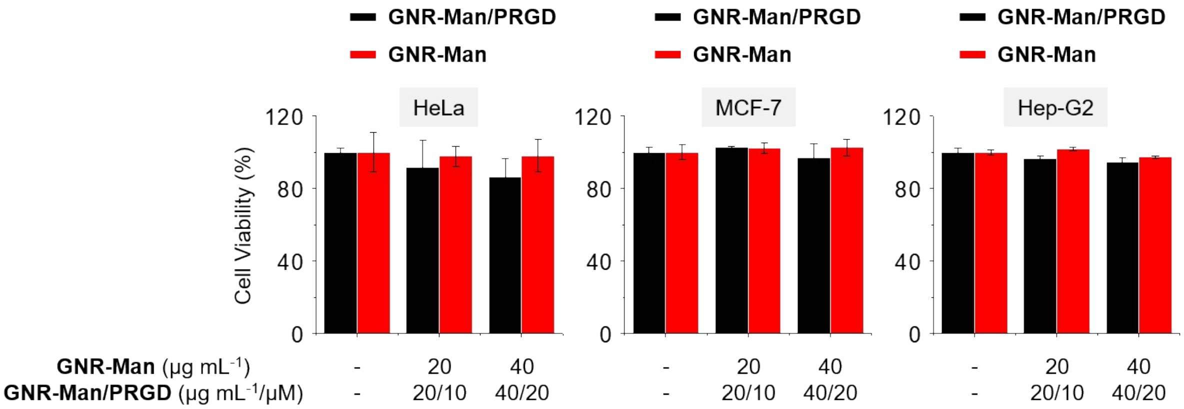

Figure S14. Cell viability of (a) Hela (human cervical cell), (b) MCF-7 (human breast cancer cell) and (c) Hep-G2 (human hepatoma cell) in the absence and presence of increasing **GNR-Man** (0, 20 and 40 μg mL^{-1}) and GNR-Man/PRGD (0/0, 20 μg mL^{-1}/10 μM and 40 μg mL^{-1}/20 μM). Cells were incubated with the materials for 30 min, and then washed. Then, cells were treated with NIR light irradiation (808 nm, 1 W cm^{-2}) for 3min prior to the cell viability assay. S. D. means standard deviation (n = 3).

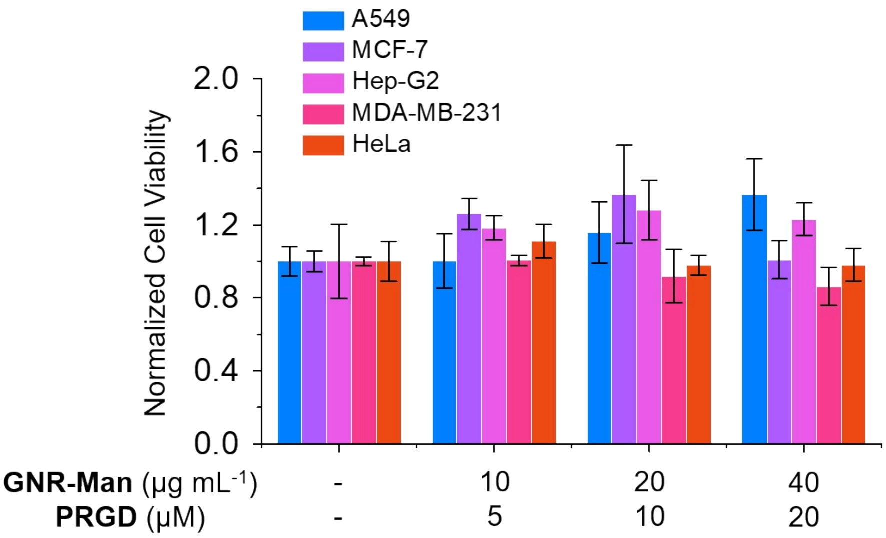

Figure S15. Cell viability of A549 (human lung cancer), MCF-7 (human breast cancer), Hep-G2 (human liver cancer), MDA-MB-231 (human triple-negative breast cancer) and HeLa (human cervical cancer) treated with increasing **GNR-Man/PRGD** (-/-, 10/5, 20/10 and 40/20 μg mL^{-1}/μM), measured by an MTS assay. All cells were treated with the material for 30 min prior to a cell viability assay for 72 hrs. S. D. means standard deviation (n = 3).

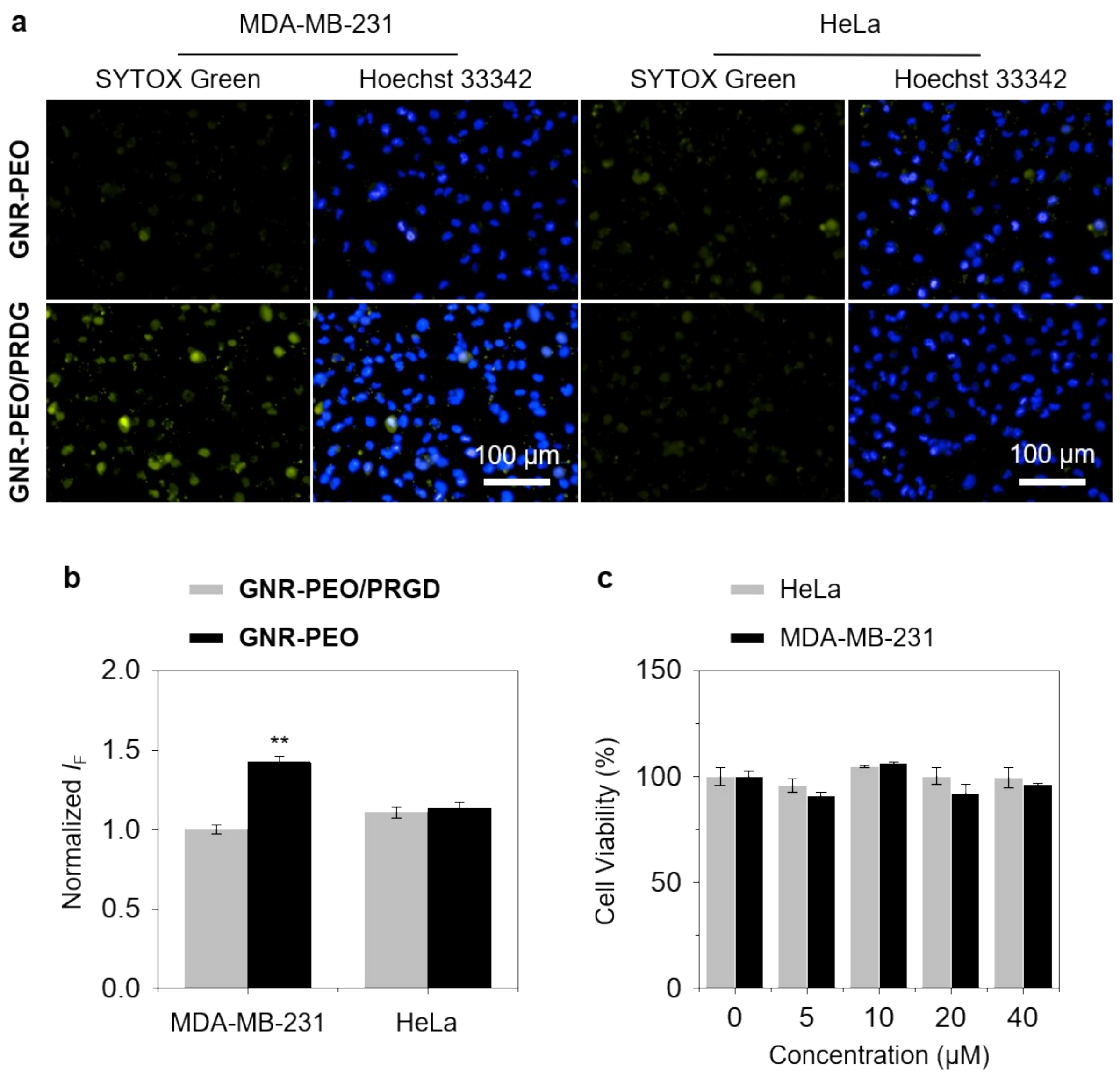

Figure S16. (a) Fluorescence imaging and (b) quantification (**$P < 0.01$ with respect to the **GNR-PEO/PRGD** group) of dead cells of MDA-MB-231 and HeLa incubated with **GNR-PEO** (40 μg mL^{-1}) in the absence and presence of **PRGD** (20 μM) under NIR light irradiation (808 nm, 1.0 W cm^{-2}) for 3 min. Dead and total cells were stained with SYTOX Green (λ_{ex} = 490-510 nm, λ_{em} = 530-590 nm) and Hoechst 33342 (λ_{ex} = 360-400 nm, λ_{em} = 410-480 nm), respectively. (c) Cell viability of MDA-MB-231 and HeLa treated with increasing **PRGD** (0, 5, 10, 20 and 40 μM), measured by an MTS assay. **PRGD** was incubated with the cells for 30 min prior to a cell viability assay for 72 hrs. S. D. means standard deviation (n = 3).

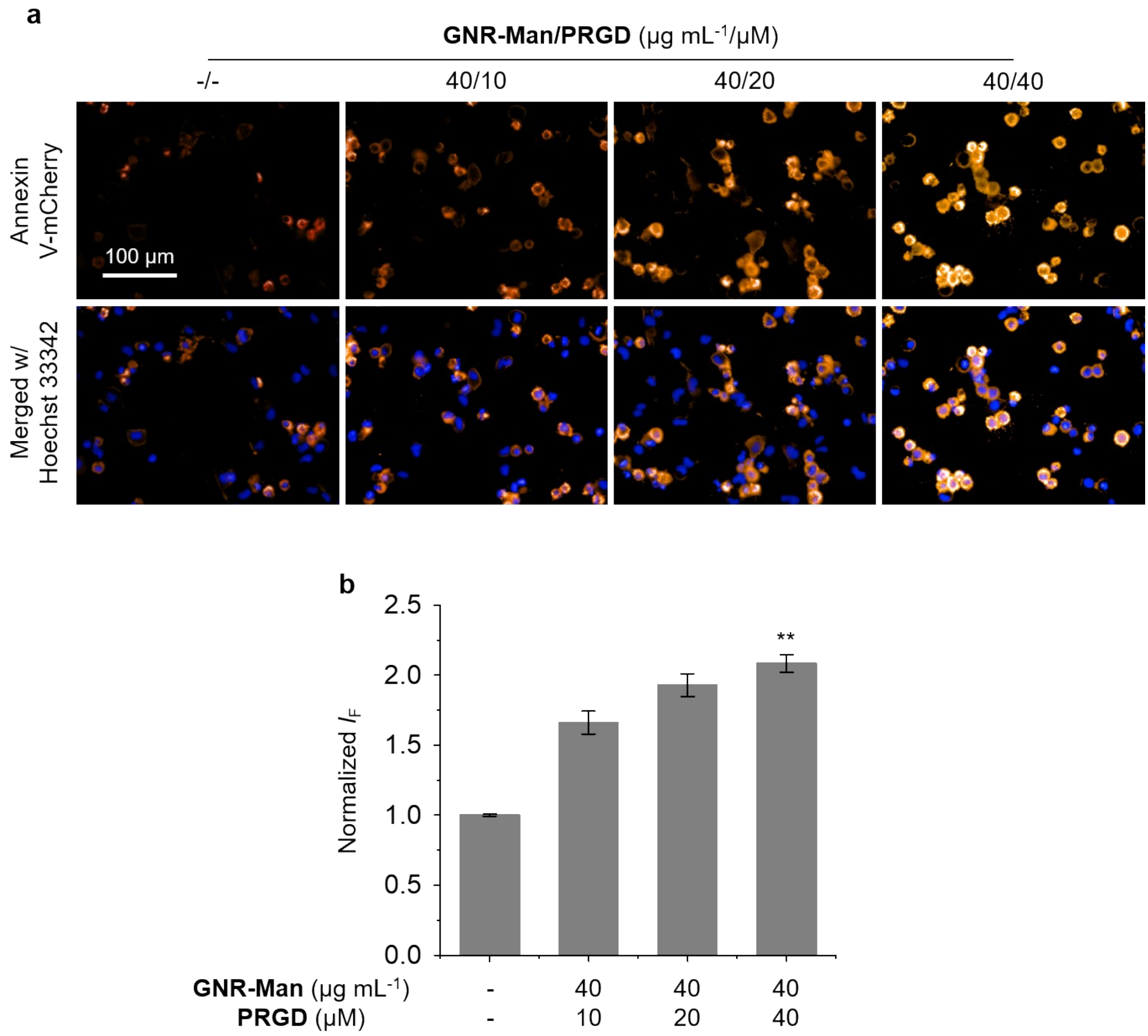

Figure S17. (a) Fluorescence imaging (b) quantification (**$P < 0.01$ with respect to the group without **PRGD**) of apoptotic MDA-MB-231 cells incubated with **GNR-Man** (40 μg mL^{-1}) with increasing of peptide **PRGD** (0, 10, 20 and 40 μM) under NIR light irradiation (808 nm, 1.0 W cm^{-2}) for 3 min. Scale bar is 100 μm, dead and total cells were stained with Annexin V-mCherry (λ_{ex} = 520-550 nm, λ_{em} = 580-650 nm) and Hoechst 33342 (λ_{ex} = 360-400 nm, λ_{em} = 410-480 nm), respectively. S. D. means standard deviation (n = 3).

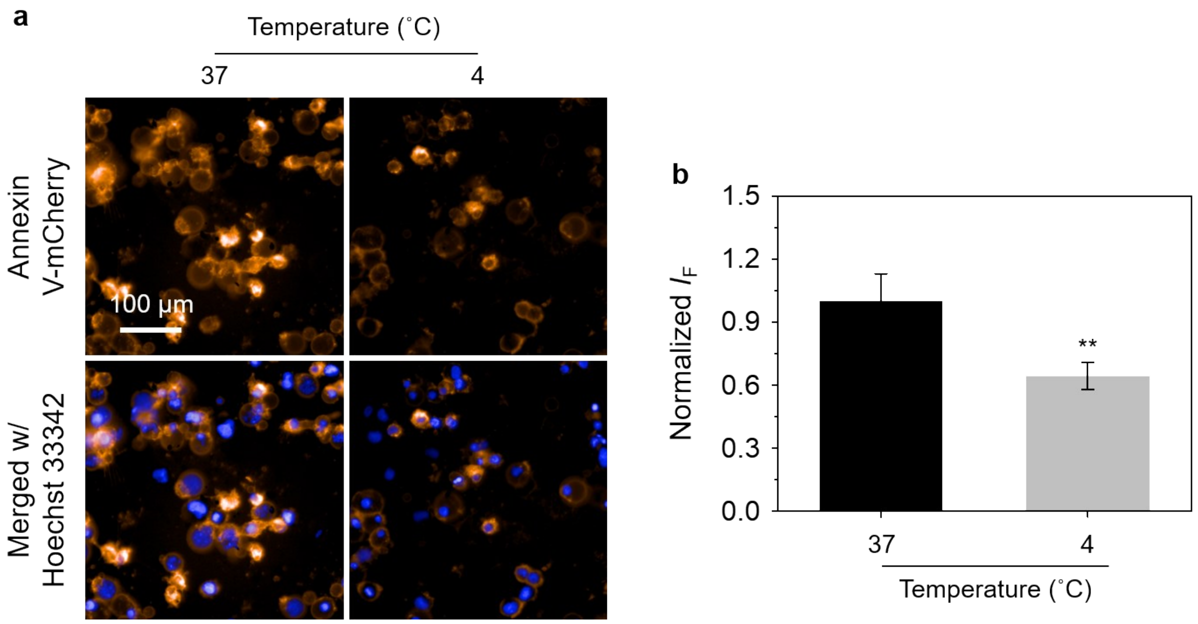

Figure S18. (a) Fluorescence imaging (b) quantification (**P < 0.01) of MDA-MB-231 incubated with **GNR-Man/PRGD** (40 µg mL^{-1}/20 µM) under NIR light irradiation (808 nm, 1.0 W cm^{-2}) for 3 min at 37 °C and 4 °C. Apoptotic and total cells were stained with Annexin V-mCherry (λ_{ex} = 520-550 nm, λ_{em} = 580-650 nm) and Hoechst 33342 (λ_{ex} = 360-400 nm, λ_{em} = 410-480 nm), respectively. S. D. means standard deviation (n = 3).

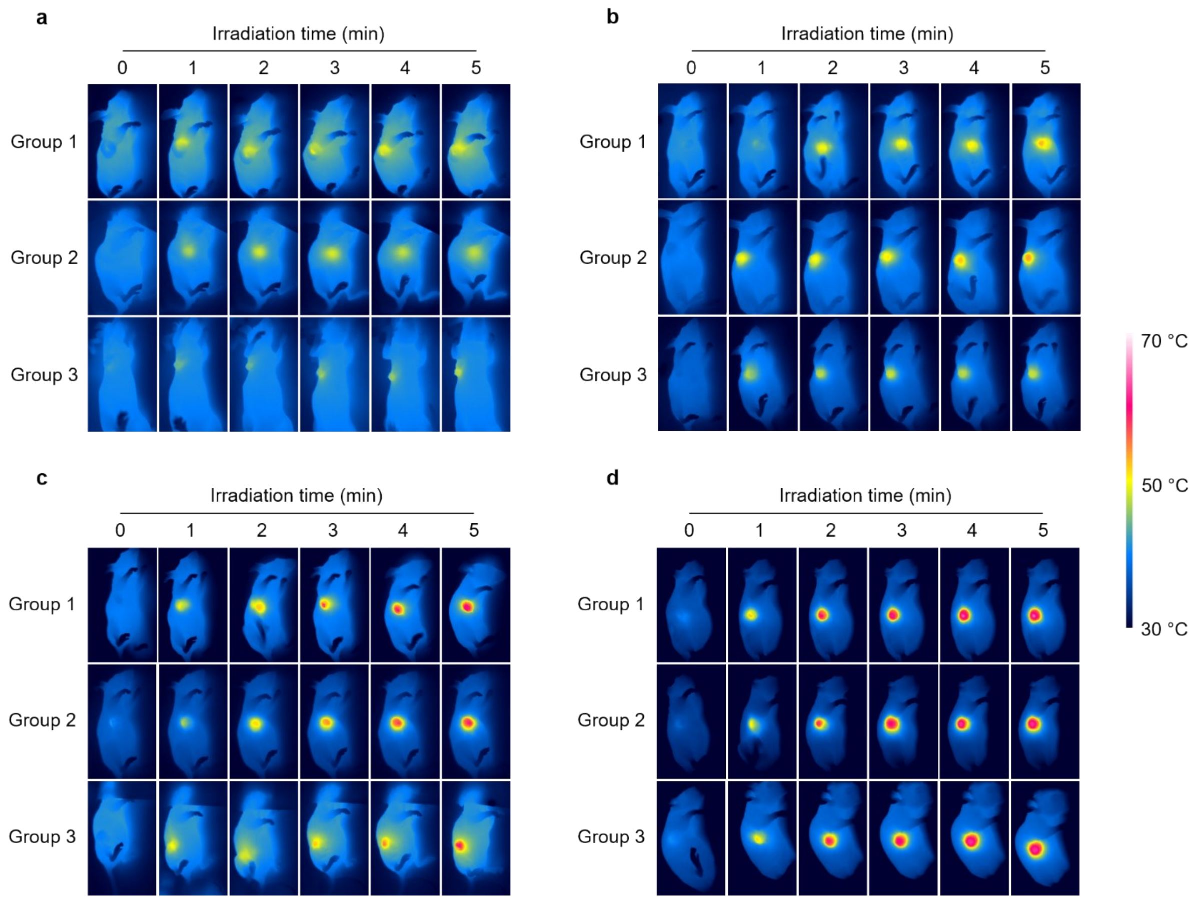

Figure S19. Photothermal images of living mice bearing MDA-MB-231-xenograft tumor under 808 nm light (1.5 W cm^{-2}) irradiation for 5 min. Photos were taken 24 h after systemic administration of PBS (0.01 M, pH 7.40), **GNR-PEO** (0.5 mg mL^{-1}), **GNR-Man** (0.5 mg mL^{-1}) and **GNR-Man/PRGD** (0.5 mg mL^{-1}/0.25 mM) through tail-vein injection (n = 3); the injection volume was 5 mg Kg^{-1}.

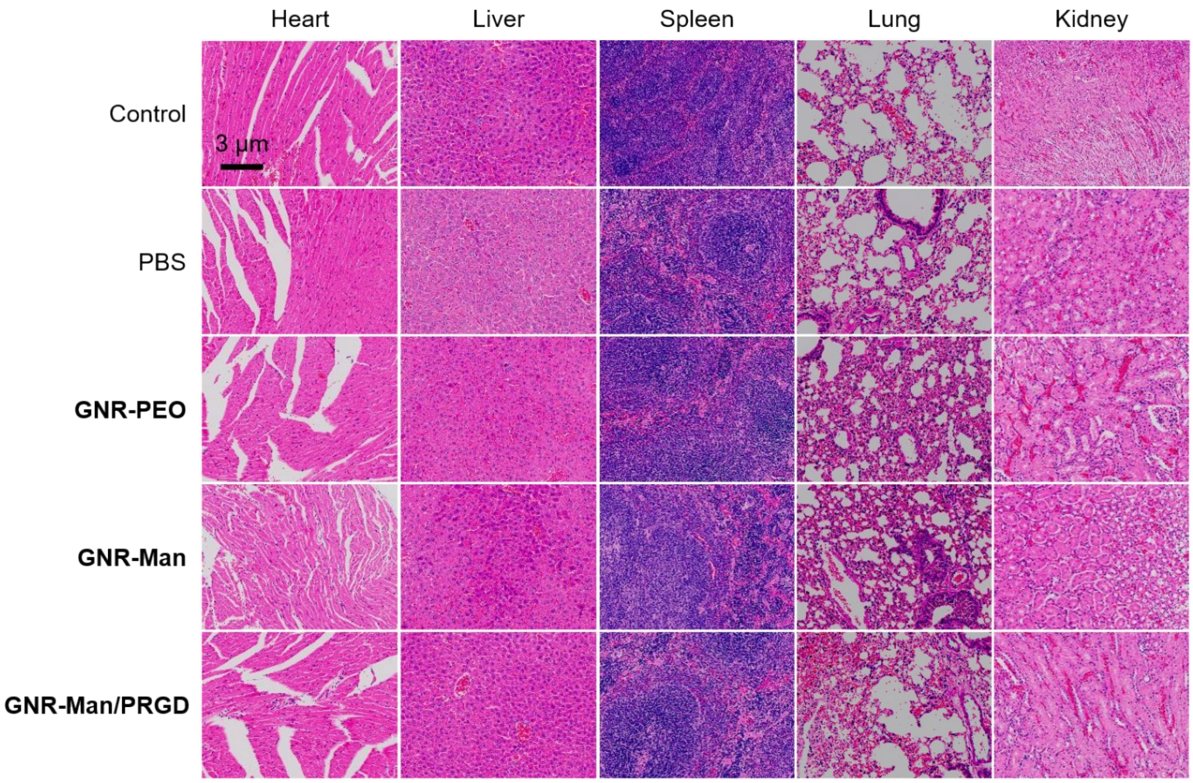

Figure S20. H&E staining of different organ slices (heart, liver, spleen, lung and kidney) removed from mice in different treatment groups after PTT for 15 days. Scale bar is applicable to all the images.

3. Spectra of new compounds

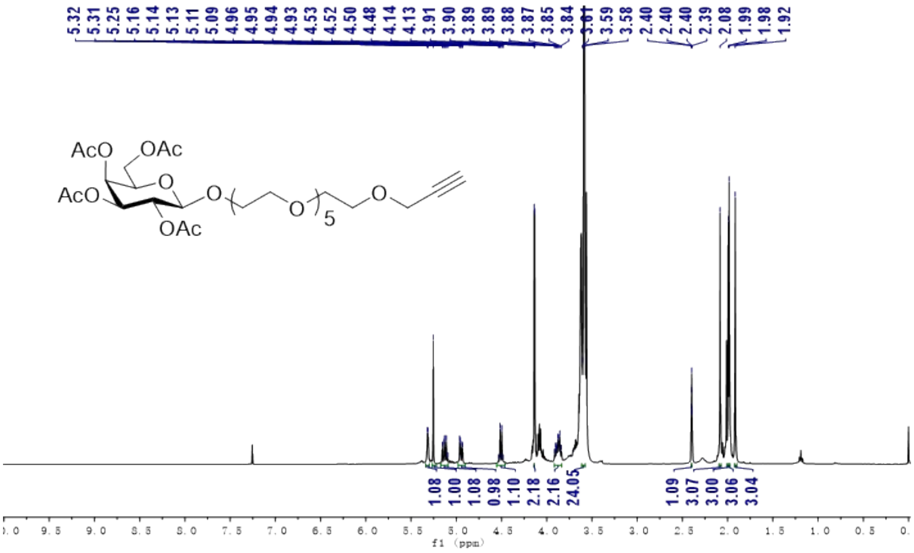

^{1}H NMR spectrum of **4**

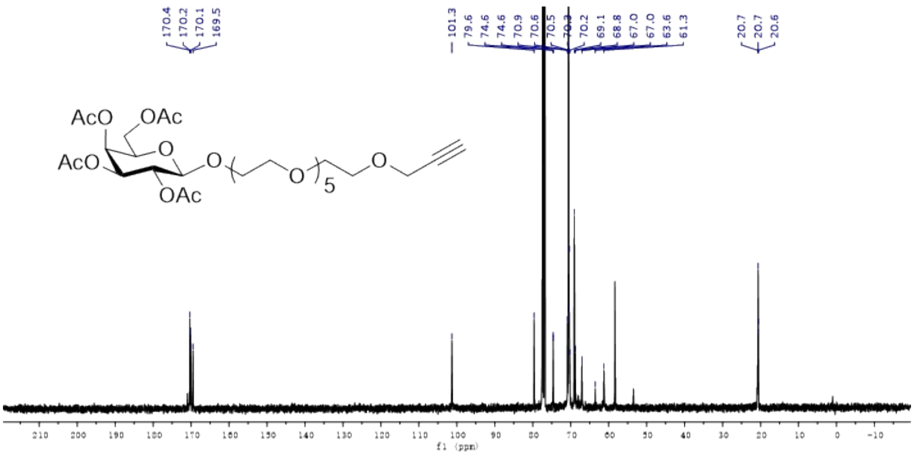

^{13}C NMR spectrum of **4**

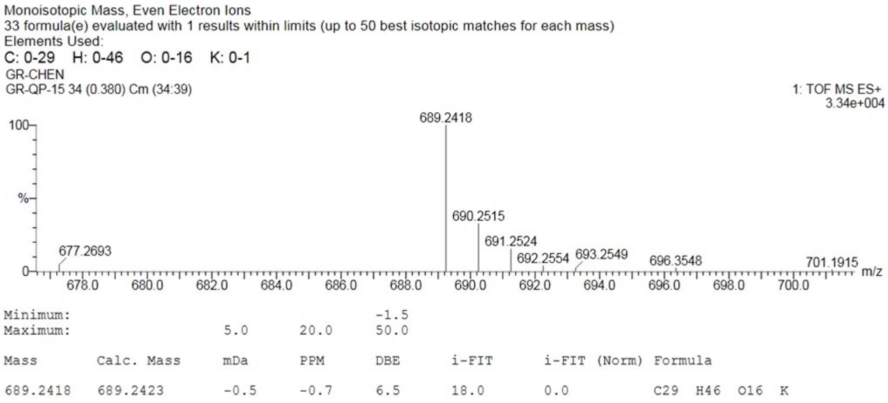

HR-ESI-MS of **4**

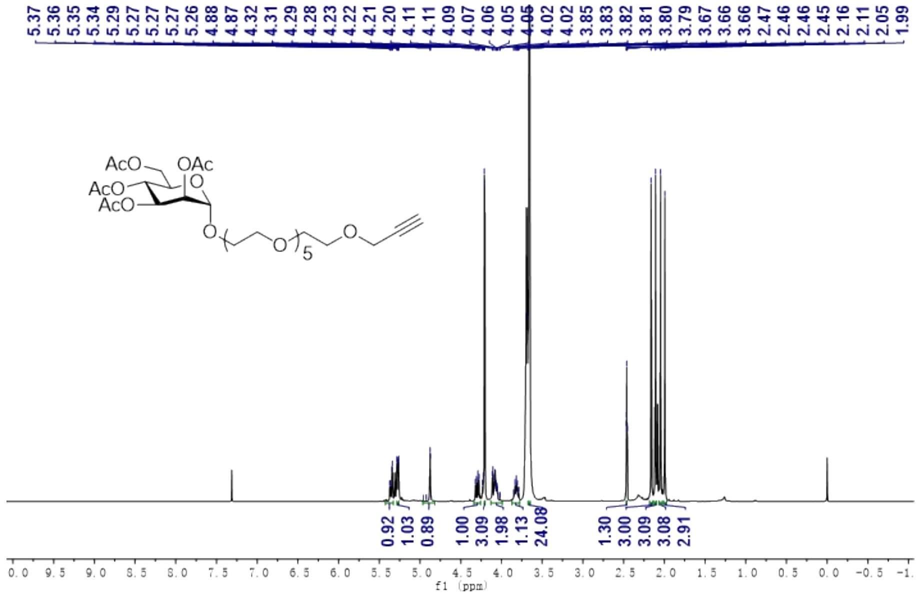

^{1}H NMR spectrum of **5**

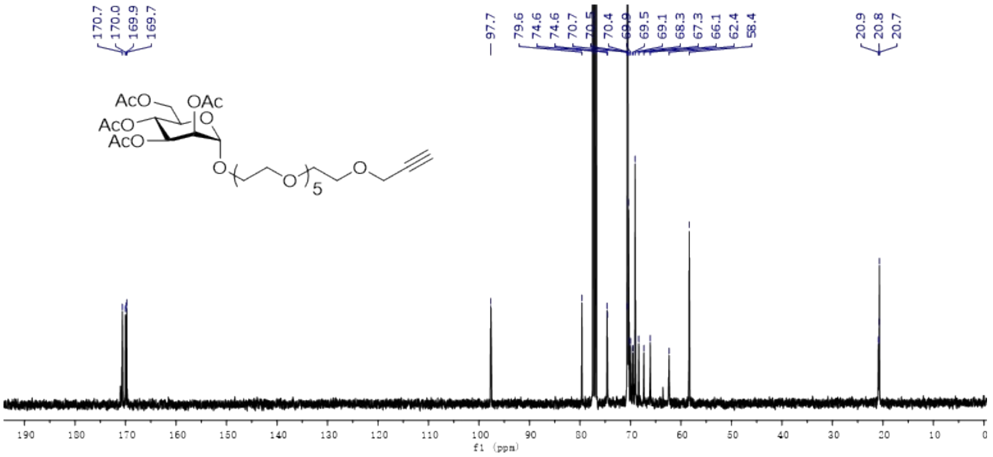

^{13}C NMR spectrum of **5**

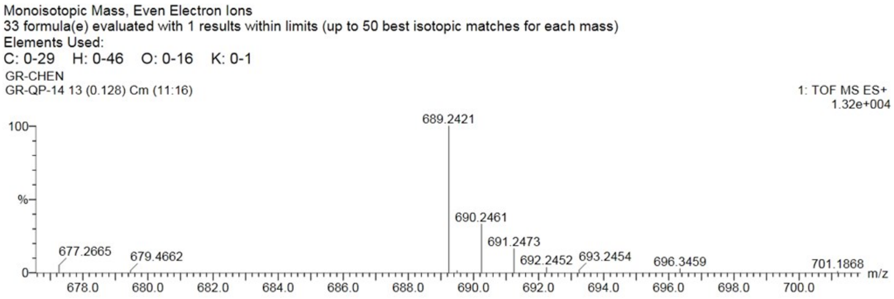

Minimum:				-1.5			
Maximum:		5.0	20.0	50.0			
Mass	Calc. Mass	mDa	PPM	DBE	i-FIT	i-FIT (Norm)	Formula
689.2421	689.2423	-0.2	-0.3	6.5	21.2	0.0	C29 H46 O16 K

HR-ESI-MS of **5**

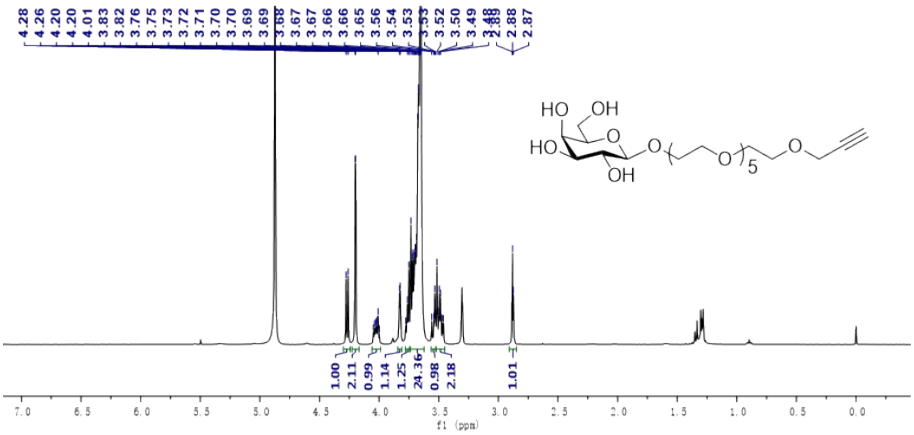

^{1}H NMR spectrum of **Alkynyl-Gal**

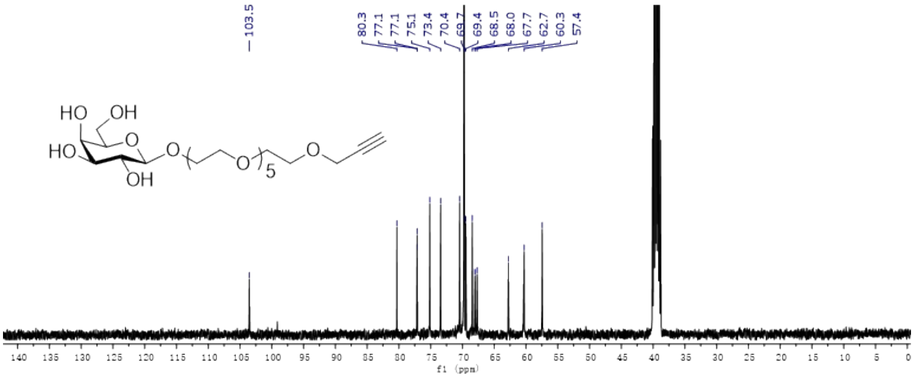

^{13}C NMR spectrum of **Alkynyl-Gal**

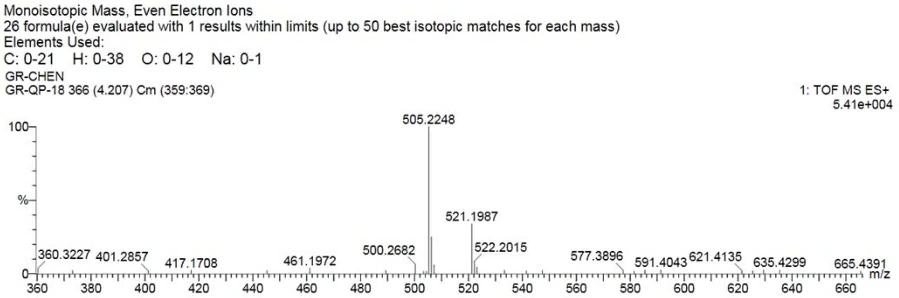

Minimum:				-1.5			
Maximum:		5.0	20.0	50.0			
Mass	Calc. Mass	mDa	PPM	DBE	i-FIT	i-FIT (Norm)	Formula
505.2248	505.2261	-1.3	-2.6	2.5	14.9	0.0	C21 H38 O12 Na

HR-ESI-MS of **Alkynyl-Gal**

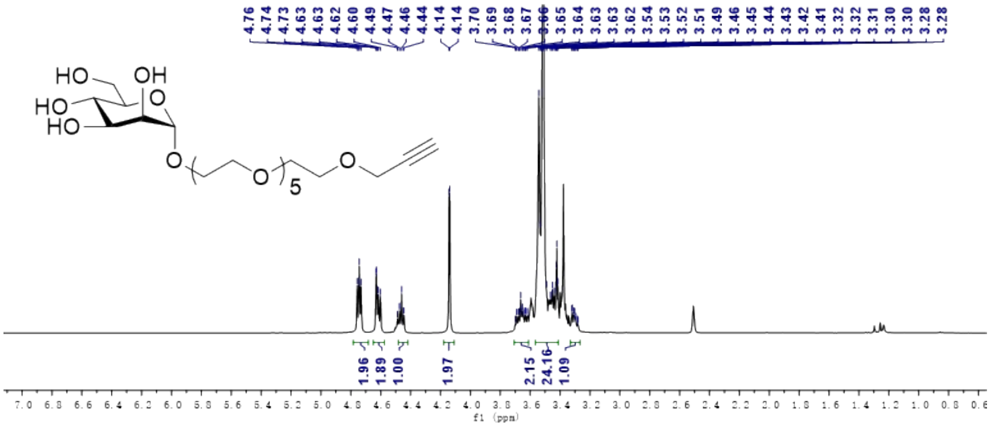

^{1}H NMR spectrum of **Alkynyl-Man**

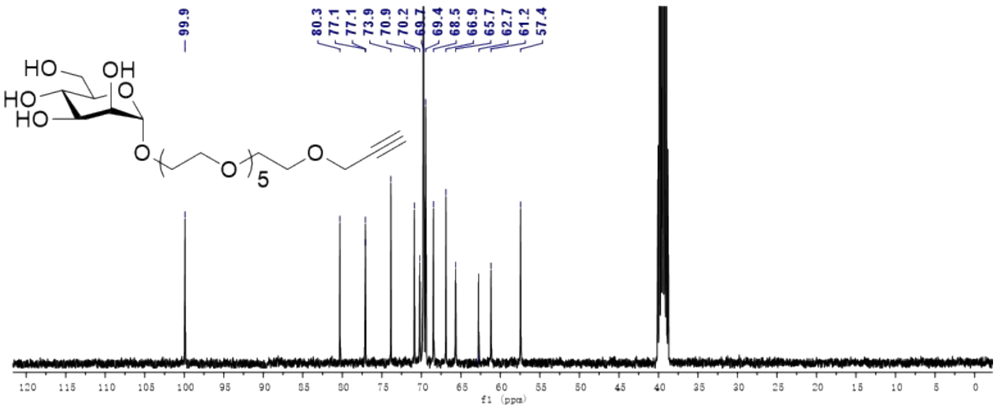

^{13}C NMR spectrum of **Alkynyl-Man**

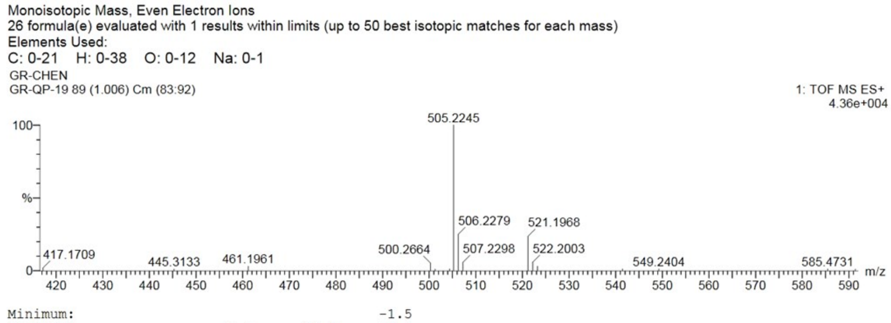

Minimum:				-1.5			
Maximum:		5.0	20.0	50.0			
Mass	Calc. Mass	mDa	PPM	DBE	i-FIT	i-FIT (Norm)	Formula
505.2245	505.2261	-1.6	-3.2	2.5	9.2	0.0	C21 H38 O12 Na

HR-ESI-MS of **Alkynyl-Man**

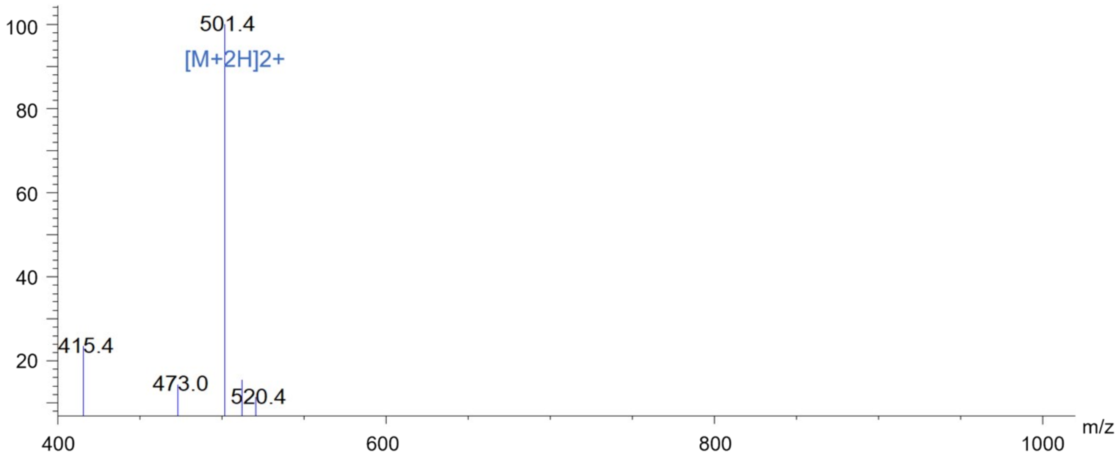

LCMS of **PRGD**

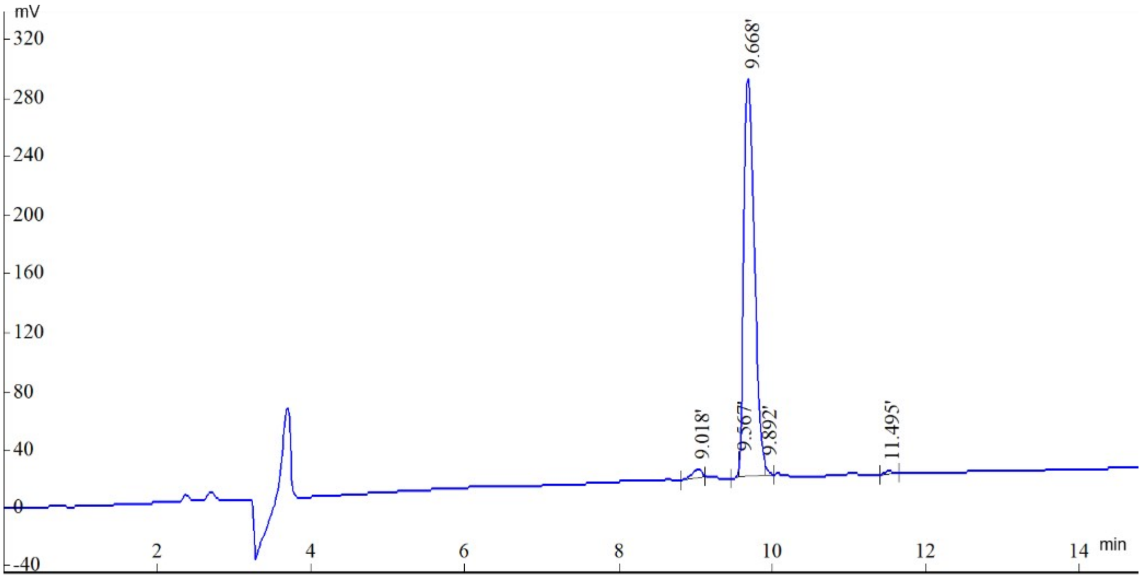

HPLC of **PRGD**

4. Additional References

[1] N. Ranjan, S. Story, G. Fulcrand, F. Leng, M. Ahmad, A. King, S. Sur, W. Wang, Y.-C. Tse-Dinh, D. P. Arya, *J. Med. Chem.* **2017**, *60*, 4904-4922.

[2] Y. Huang, W.-T. Dou, F. Xu, H.-B. Ru, Q. Gong, D. Wu, D. Yan, H. Tian, X.-P. He, Y. Mai, X. Feng, *Angew. Chem. Int. Ed*. **2018**, *57*, 3366-3371.

[3] H. Lin, S. Gao, C. Dai, Y. Chen, J. Shi, *J. Am. Chem. Soc.* **2017**, *139*, 16235-16247.

[4] F.Xu, H. Li, Q. Yao, H. Ge, J. Fan, W. Sun, J.Wang, X. Peng, *Chem. Sci*. **2019**, *10*, 10586-10594.
